# A systematic review and meta-analysis of the evidence on the acute effects of caffeine on sport-specific skills, physical performance, and physiological function in female basketball players

**DOI:** 10.3389/fnut.2026.1766993

**Published:** 2026-02-23

**Authors:** Zike Zhang, Mingyue Yin, Bopeng Qiu, Fanhao Meng, Bitai Wu, Yimin Wang, Zimao Cheng, Xiaolong Wang, Youheng Wang, Zhe Lu, Yunxiang Sun, Jiali Lai

**Affiliations:** 1 School of Physical Education, Shaanxi Normal University, Xi'an, China; 2 School of Competitive Sports, Shanghai University of Sport, Shanghai, China; 3 Division of Sports Science & Physical Education, Tsinghua University, Beijing, China; 4 School of Strength and Conditioning Training, Beijing Sport University, Beijing, China; 5 School of Physical Education, Changsha University of Science and Technology, Changsha, Hunan, China

**Keywords:** basketball, caffeine, ergogenic aids, female athletes, sports performance

## Abstract

**Introduction:**

Previous original research and meta-analyses have shown that caffeine enhances performance in basketball. However, most studies on caffeine’s effects in basketball have focused on male or mixed-gender samples, and there is currently no meta-analysis specifically on caffeine’s impact on female basketball performance. This study aimed to synthesize current evidence to examine the effects of caffeine supplementation on multidimensional performance outcomes in female basketball players.

**Methods:**

A systematic search was conducted in May 2025 across PubMed, Web of Science, Embase, Scopus, the Cochrane Library, ProQuest, and EBSCO. Randomized crossover trials examining the effects of caffeine intake on performance in female basketball players were included. Methodological quality and risk of bias were assessed using the PEDro scale and the RoB 2 tool, and the certainty of evidence was evaluated using the GRADE approach.

**Results:**

Seven studies met the inclusion criteria, demonstrating overall high methodological quality and low risk of bias. The meta-analysis showed that, in female basketball players, caffeine intake may have a moderate effect on power output, whereas it did not produce statistically significant effects on sport-specific skills (shooting accuracy and dribble sprint performance), physical performance (jump height, agility, off-ball sprinting, and anaerobic capacity), or physiological function (perceived fatigue and physiological/biochemical markers) in female basketball players. Caffeine consumption was, however, associated with potential adverse effects, including insomnia, tachycardia, and headache. Although statistical significance was not reached for several outcomes, moderate effect sizes were observed for jump height (*p* = 0.08), off-ball sprint performance (*p* = 0.051), and physiological/biochemical markers (*p* = 0.052), suggesting that caffeine may exert a potential modulatory effect on performance.

**Conclusion:**

Across the included studies, which examined caffeine doses ranging from 2.1 to 9 mg/kg this review found no statistically significant effects on most performance outcomes in female basketball players, although a significant pooled effect was observed for power output. However, effect sizes varied across performance measures and may be modulated by menstrual cycle–related physiological factors. Given the intermittent demands of basketball and the limited sample sizes of available studies, further research with larger cohorts and more rigorous study designs is warranted.

**Systematic review registration:**

https://www.crd.york.ac.uk/prospero/, PROSPERO: CRD420251025291.

## 1 Introduction

Basketball is an intermittent high-intensity sport that places extremely high demands on athletes’ physiological capacities (1). During basketball games, female athletes spend approximately 10 min performing high-intensity actions with and without the ball, such as jumping and sprinting (2, 3). Specifically, about 57% of sprints performed by female players occur over distances of 1–5 m, while 30% cover distances of 6–10 m. In addition, 52% of sprints involve curvilinear movement patterns and changes in direction (3), and 76% of fast-break advances are completed through dribbling sprints (4). On average, players perform more than 50 jumping actions per game, and 48.7% of basketball activities involve combined techniques of jumping and shooting (5, 6). Moreover, shooting accuracy is a critical determinant of game outcomes (7). It is evident that many key actions performed during basketball training and competition are based on vertical movements (e.g., rebounding and jump shooting), and horizontal movements (e.g., changes of direction and sprinting) (8). Overall, these characteristics impose extremely high internal and external loads on athletes (1), requiring female players to possess well-developed anaerobic capacity and explosive power as fundamental physical qualities (9). Such dense and irregular high-load demands impose substantial stress on the cardiovascular and neuromuscular systems. Without adequate regulation, cumulative fatigue may develop, leading to declines in performance and an elevated risk of injury (10, 11). To meet the physiological challenges of training and competition, athletes commonly rely on targeted nutritional strategies to support functional capacity and recovery (12). Among available ergogenic aids, caffeine has become one of the most frequently used due to its demonstrated ability to enhance endurance and performance across various sport modalities (13, 14).

Caffeine (1,3,7-trimethylxanthine) is a widely consumed psychoactive compound, and since its removal from the World Anti-Doping Agency (WADA) Prohibited List in 2004, it has been widely used by athletes for its potential ergogenic effects (15–17). Current evidence suggests that these benefits are largely attributable to adenosine receptor antagonism, which attenuates adenosine-mediated central inhibition and thereby supports alertness and fatigue resistance (18). In addition, caffeine may modulate catecholamine release (19) and by promoting Ca^2+^ release from the sarcoplasmic reticulum, contribute to greater force production (20). Collectively, these central and peripheral actions may enhance motor-unit recruitment and firing frequency in large muscle groups, translating into improved strength- and endurance-related performance (21, 22). Consistent with published consensus position statements, the International Society of Sports Nutrition (ISSN) classifies caffeine as an ergogenic aid with relatively robust evidence and an acceptable safety profile when used within recommended doses (23).

A substantial body of research has examined the effects of caffeine on basketball performance, with most studies reporting beneficial outcomes across a range of sport-specific and physical performance measures (24–26), and such effects may vary depending on the form of caffeine intake (27). However, the available evidence is marked by a pronounced sex imbalance: investigations have largely focused on male or mixed-sex cohorts, while data specific to female athletes remain sparse. According to published estimates, women (including those in mixed-sex samples) account for only ~13% of participants in caffeine–basketball studies (28). Even when female players are included, potential sex-related differences are rarely evaluated in a systematic manner (24, 29, 30). Given this scarcity of female-specific research, and considering that fluctuations in estrogen and progesterone across the menstrual cycle may influence exercise performance in female basketball players (31), as well as the fact that oral contraceptive use may prolong caffeine half-life and delay its metabolic clearance in women (32), the direct extrapolation of findings from male samples to women constitutes a clear limitation and raises uncertainty about real-world applicability in female basketball (33, 34). Multiple authors have therefore highlighted the need for dedicated studies in women, underscoring a persistent gap in the literature (35–37). In line with this context, Gomez-Bruton et al. (28) recently published a systematic review of caffeine use in female team-sport athletes. Although basketball was included in this review, considering the scope of the databases searched and the research focus, which primarily targeted female athletes participating in team sports, most of the included studies involved mixed samples of female athletes from multiple sports or combined male and female participants, among which only two original studies (2, 38) directly investigated female basketball players. Consequently, the current body of evidence remains insufficient to support the development of reliable and practically meaningful recommendations specifically for female basketball players (28).

Given these limitations, the effects of caffeine on the performance of female basketball players remain to be clarified. To provide a comprehensive understanding of caffeine’s effects, we have classified performance outcomes into three distinct domains: sport-specific skills, physical performance, and physiological function. This grouping allows for a clear distinction between technical skills, overall physical capacity, and internal physiological responses, ensuring a thorough evaluation of caffeine’s impact on both external performance and underlying biological processes. Accordingly, a systematic review and meta-analysis was conducted to systematically evaluate the acute influence of caffeine supplementation on sport-specific skills (e.g., shooting accuracy and dribbling speed performance), physical performance (e.g., jump height, sprint and agility performance), and physiological function (e.g., Fatigue-Perception Performance, physiological and biochemical markers) in female basketball athletes. By expanding the scope of database searches, conducting additional reference tracking, and specifically extracting data pertaining only to female basketball players, this study included a total of seven original studies. The aim of this study was to the evidence regarding the acute effects of caffeine on sport-specific skills, physical performance, and physiological function in female basketball players, and to examine the potential moderating roles of menstrual cycle phase, caffeine dose, and timing of intake.

## 2 Materials and methods

### 2.1 Search strategy

This systematic review and meta-analysis was conducted in accordance with the Preferred Reporting Items for Systematic Reviews and Meta-Analyses (PRISMA) 2020 guidelines (Supplementary Table 1) (39) and was pre-registered in PROSPERO (CRD420251025291). A systematic search was conducted in PubMed, Web of Science, Embase, Scopus, the Cochrane Library, ProQuest, and EBSCO from inception to May 1, 2025. Search strategies were adapted for each database using Boolean operators and wildcards. The detailed search strategies for each database are provided in Supplementary Table 2. All retrieved records were exported to a CSV file for deduplication in EndNote X9 (Clarivate Analytics, New York, NY, United States). Study identification, screening, and final selection were independently completed by two reviewers (ZZK and YMY), with disagreements resolved through discussion.

### 2.2 Inclusion and exclusion criteria

The inclusion and exclusion criteria were defined according to the PICOS framework (Supplementary Table 3) as follows: (1) Participants were adult female basketball players (18 years of age or over) across different training and competitive levels. In studies including both sexes, only data from female participants were extracted; when unavailable, corresponding authors were contacted for data acquisition (4, 24, 30), studies were excluded if the required data remained unobtainable after contact (40). Studies involving para-athletes were also excluded; (2) Interventions examined the acute effects of caffeine. Longitudinal studies assessing chronic caffeine intake, trials involving multi-ingredient supplements in which caffeine was not the primary active component, and studies lacking a clearly documented caffeine supplementation protocol were excluded; (3) Studies that used a design including a placebo condition as a comparator to caffeine; (4) crossover studies that compared the intake of caffeine and a placebo; (5) Studies using a blinded and randomized design; (6) Studies reporting at least one measure of athletic performance (e.g., vertical jump, physiological indicators). Unpublished data, grey literature, systematic reviews, and meta-analyses were excluded.

### 2.3 Data extraction and transformation

Data extraction was independently conducted by two authors (ZZK and QBP) using a customized Excel form developed prior to full-text screening. Information regarding menstrual cycle phase and oral contraceptive use was extracted when reported. For studies with missing data or outcomes reported exclusively in graphical format, corresponding authors were contacted via email to obtain the required information. If no response was received and the necessary data were available in figures, numerical values were extracted using WebPlotDigitizer 4.1. All digitized data were independently verified by two researchers, with any discrepancies resolved through consensus.

In this systematic review, for studies reporting post-intervention group means and standard deviations (caffeine vs. placebo), these values were directly extracted to calculate the standardized mean difference (SMD). When outcomes were reported only as pre–post measurements, baseline and final measurements were extracted to derive the mean change using the following equation (41):


$$M_{\text{diff}}=M_{\text{post}}-M_{\mathrm{pre}}$$

where $M_{\text{diff}}$ denotes the mean change, $M_{\text{post}}$ the reported post-intervention mean, and $M_{\mathrm{pre}}$ the reported pre-intervention mean.

The standard deviation of the mean change ( $SD_{\text{diff}}$ ) was then calculated as follows (41):


$$SD_{\text{diff}}=\sqrt{S{D_{\mathrm{pre}}}^{2}+S{D_{\text{post}}}^{2}-2r\times SD_{\mathrm{pre}}\times SD_{\text{post}}}$$

Where r represents the pre–post correlation coefficient. In accordance with the Cochrane Handbook, a conservative value of 0.5 was adopted in this study (41). The calculated mean change and its standard deviation were subsequently used to compute the standardized mean difference (SMD) for the meta-analysis.

### 2.4 Quality assessment of included studies

The methodological quality of the included studies was assessed using the PEDro scale. Based on total scores, studies were classified as excellent (9–10), good (6–8), fair (4–5), or poor (<4). The PEDro scale has demonstrated good reliability and validity for evaluating the internal validity of randomized controlled trials (42).

Risk of bias was assessed using the RoB 2 tool in accordance with Cochrane guidelines (43). The evaluation encompassed the following domains: (1) bias arising from the randomization process; (2) bias due to deviations from intended interventions; (3) bias due to missing outcome data; (4) bias in outcome measurement; and (5) bias in selection of the reported results. For crossover trials, period and carryover effects were additionally examined as potential sources of bias. Each study was rated as having low risk, some concerns, or high risk of bias.

Both the PEDro and RoB2 tools were applied by two independent researchers (ZZK and MFH), with any disagreement resolved through consensus (Supplementary Table 4).

### 2.5 Evidence grading

The certainty of evidence was assessed using the GRADE approach and categorized as high, moderate, low, or very low (44). The evaluation followed structured criteria: (1) Risk of bias: The certainty was downgraded by one level for outcomes rated as having “some concerns,” and by two levels for those rated as “high risk.” (2) Inconsistency: The certainty was downgraded by one level when heterogeneity fell between 25 and 75% (I^2^ = 25–75%), and by two levels when heterogeneity exceeded 75%. (3) Imprecision: The certainty was downgraded by one level when the results lacked statistical significance. (4) Publication bias: In accordance with the Cochrane Hand-book (45), formal assessments of publication bias (e.g., Egger’s test) were not conducted due to the limited number of included studies (<10). Consequently, the certainty of evidence was not downgraded for publication bias. However, we acknowledge that the small number of included studies limits the statistical power to detect potential publication bias, which should be considered a limitation of this review rather than evidence of its absence (Supplementary Table 5).

Given the limited and inconsistent control of menstrual cycle phase and the lack of reporting on oral contraceptive use across studies, these factors were prespecified as sources of physiological heterogeneity and were considered during qualitative data synthesis and interpretation. This limitation also informed the cautious evaluation of the certainty of evidence.

### 2.6 Statistical analysis

Data synthesis was conducted using the inverse-variance method under a random-effects model, implemented with the Der Simonian–Laird estimator (42). Between-study variance components (tau^2^ and tau) and their confidence intervals were estimated using the Jackson method (44). Pooled effect sizes were calculated using standardized mean differences (SMD), and their corresponding 95% confidence intervals (95% CI). Given the generally small sample sizes across the included studies, the bias-corrected Hedges’ g was used as the primary effect size metric (hereafter denoted as ES), with interpretive thresholds defined as follows: <0.2 trivial, 0.2–0.5 small, 0.5–0.8 moderate, and >0.8 large (46).

Heterogeneity was assessed using the I^2^ statistic, tau^2^, and tau, and a prediction interval (PI) was calculated to estimate the potential range of true effects in comparable future studies (47). I^2^ values were interpreted as 0–25% low, 25–75% moderate, and >75% high heterogeneity (48). Publication bias was evaluated via funnel plots and Egger’s test (49, 50) with *p* > 0.05 indicating no significant bias.

All statistical analyses and graphical outputs were performed in RStudio (v4.5.0) using the meta and metafor packages. Statistical significance was set at *p* < 0.05, while *p*-values between 0.05 and 0.10 were considered indicative of a potential trend. Because standardized mean differences are sensitive to very small within-group variability, particularly in single-study subgroups (k = 1), results from such subgroups were interpreted with caution and used for descriptive purposes only.

Leave-one-out sensitivity analyses were conducted for outcomes including three or more studies by iteratively removing one study at a time and re-running the meta-analysis to assess the influence of individual studies on the pooled effect estimates. For outcomes supported by only two studies, formal sensitivity analyses were not feasible. Therefore, the robustness of these findings should be interpreted with caution (51). Prespecified sensitivity analyses, defined *a priori* based on the RoB 2 risk-of-bias assessment, were conducted by excluding studies with concerns in Domain 5 (bias in selection of reported results) to assess whether pooled effect estimates were driven by these studies.

## 3 Results

### 3.1 Literature screening process

**Figure 1:**
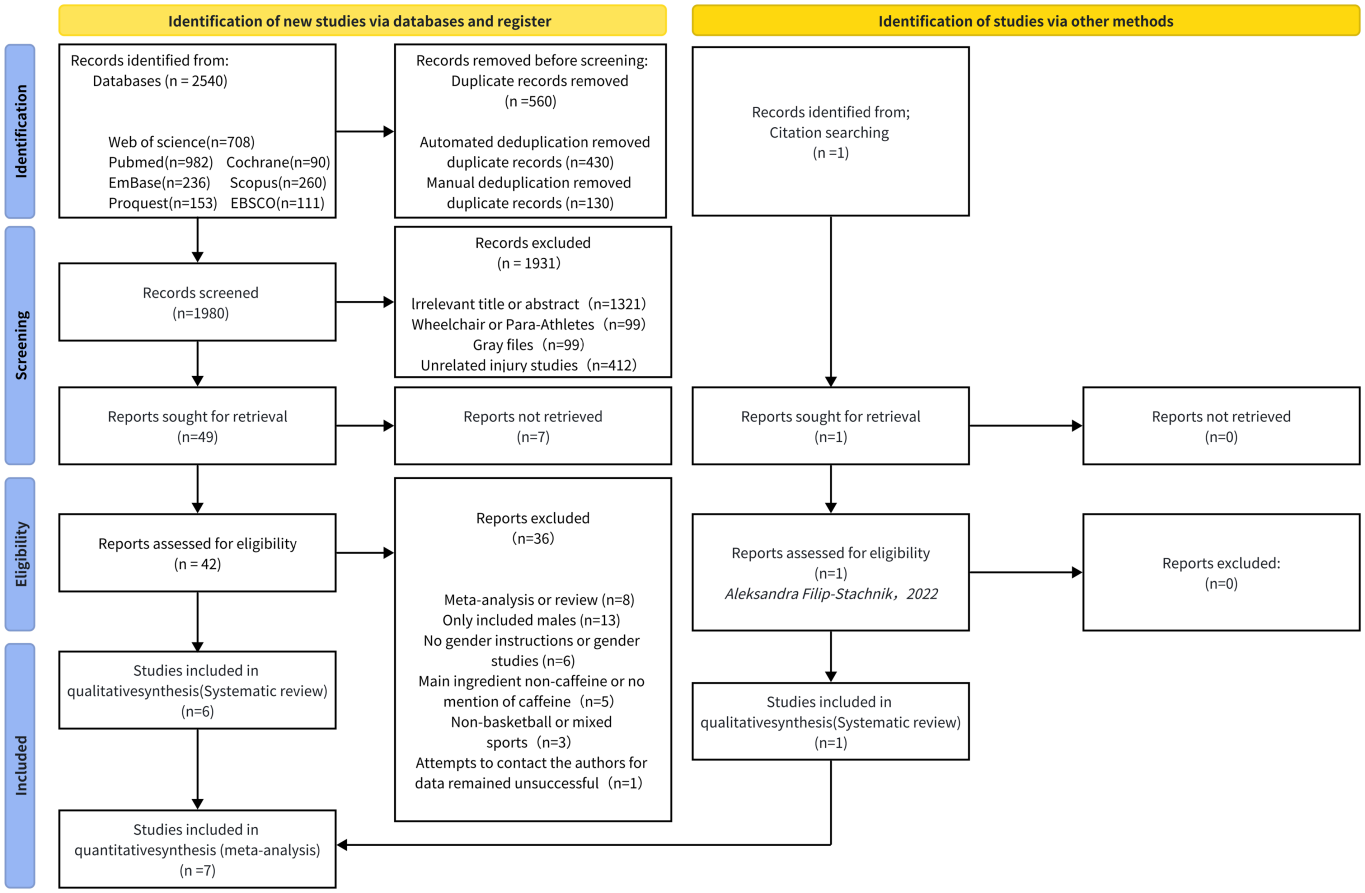
PRISMA flowchart of search strategy.

A systematic search of six electronic databases, including PubMed (*n* = 982), Web of Science (*n* = 708), EMBASE (*n* = 236), Scopus (*n* = 260), ProQuest (*n* = 153), and EBSCO-host (*n* = 111) and the Cochrane Library (*n* = 90), yielded a total of 2,540 records. One additional study was identified through manual searching of reference lists (52). After removing duplicates and screening for eligibility, seven studies met the inclusion criteria (2, 4, 38, 52–55) (Figure 1), comprising 101 female basketball players.

As summarized in Table 1, the general characteristics of the included studies cover: (1) Author (s), year, and country; (2) study design; (3) Participants’ characteristics (sample size and, where reported, training level, age, body weight, height, menstrual cycle phase); (4) Caffeine consumption or restrictions; (5) Intervention (form, timing, and dosage); (6) Comparator (s) (form, dosage, and protocol); (7) Study period and washout; (8) Performance and physiological outcomes; (9) Funding source.

**Table 1:** Characteristics of included studies.

Author(s), year, and country	Study design	Participants’ characteristics	Caffeine consumption or restrictions	Intervention (form, dosage, and timing)	Comparator(s) (form, dosage, and protocol)	Study period and washout	Performance and physiological outcomes	Funding source
Mahdavi et al. (2012) (55), Iran	Double-blind, RCT randomised controlled trial	*N* = 26 Training level: not specified Age = 24.22 ± 2.65 Y BM = 57.33 ± 6.97 kg Height = 1.643 ± 5.45 M Menstrual cycle = on day 10 of menstrual cycle	116.88 mg.day^−1^	Caffeine capsule 5 mg.kg^−1^ 70 min pre-test	Dextrose capsules 5 mg.kg^−1^ Same scheme	1 day each trial/7 days apart	Malondialdehyde Antioxidant capacity Creatine kinase White blood cells Lymphocyte Granulocyte	Nutrition Research Center of Tabriz University of Medical Sciences
Mahdavi et al. (2015) (38), Iran	Double-blind, RCT randomised controlled trial	*N* = 24 Training level: Not specified Age = 24.2 ± 2.6 Y BM = 57.3 ± 6.9 kg Height = 1.6 ± 5.45 M Menstrual cycle = Not specified	116.88–26.73 mg/day	Caffeine capsule 5 mg.kg^−1^ 70 min pre-test	Dextrose capsules 5 mg.kg^−1^ Same scheme	1 day each trial/7 days apart	Anaerobic Power Rating of Perceived Exertion (RPE) Blood Lactate Levels	Nutritional Research Center of Tabriz University of Medical Sciences
Scanlan et al. (4), Australia	Double-blind, RCT randomised controlled trial	*N* = 10 Training level: Elite players Age = 18.3 ± 3.3 Y Menstrual cycle = luteal phase	<100 mg·day^−1^	Caffeine capsule 3 mg/kg 60 min pre-test	Dextrose capsule 3 mg/kg Same scheme	1 day each trial/7 days apart	Dribbling	NR
Stojanović et al. (2019) (2), Serbia	Double-blind, RCT randomised controlled trial	*N* = 10 Training level: Professional players Age = 20.2 ± 3.9 Y BM = 69.2 ± 6.3 kg Height = 175.4 ± 5.9CM Menstrual cycle = luteal phase	<100 mg/day	Caffeine capsule 3 mg/kg 60 min pre-test	Dextrose capsules 3 mg/kg Same scheme	1 day each trial/7 days apart	Dribbling Jump Agility/Reaction Sprint Anaerobic Power Rating of Perceived Exertion (RPE) Side effects	Ministry of Education, Science, and Technological Development within the project ОI179019.
Filip-Stachnik et al. (2022) (52), Poland	Double-blind, RCT randomised controlled trial	*N* = 9 Training level: Elite players Age = 24 ± 4 Y BM = 66.8 ± 7.4 kg Height = 174 ± 4CM Menstrual cycle = Not controlled	Not restricted	Caffeine gum 2.3 ± 0.2 mg/kg 15 min pre-test	Gum 0 mg/kg caffeine same scheme	1 day each trial/7 days apart	Shot accuracy Jump Agility/Reaction Sprint Rating of Perceived Exertion (RPE)	NR
Quan et al. (2025) (54), China	Single-blind, RCT randomised controlled trial	*N* = 10 Training level: experience players Age = 23.20 ± 2.53 Y BM = 65.45 ± 4.18 kg Height = 175.39 ± 7.19CM Menstrual cycle = Not controlled	No chronic caffeine use habits	Caffeine capsule (3 mg/kg; 6 mg/kg; 9 mg/kg) 60 min pre-test	Calcium carbonate capsule 9 mg/kg Same scheme	1 day each trial/7 days apart	Jump Agility/reaction Power output Cells/Material energy	NR
Nieto-Acevedo et al. (2025) (53), Spain	Double-blind, RCT randomised controlled trial	*N* = 12 Training level: >10 /semi-professional players Age = 21.6 ± 3.8 Y BM = 68.8 ± 16.9 kg Height = 174.6 ± 9.8CM Menstrual cycle = Not controlled	<100 mg/day	Caffeine capsule 3 mg/kg 60 min pre-test	Cellulose capsules 3 mg/kg Same scheme	1 day each trial/7 days apart	Shot accuracy Jump Sprint Agility/Reaction Side effects	NR

*N* = Total number of participants; Y=Year; BM = Body Mass; NR = Not Reported; Oral contraceptive: None of the studies reported information regarding oral contraceptive use.

### 3.2 Risk of bias assessment in included studies

The PEDro scores of the included studies ranged from 7 to 10. Three studies were rated as excellent, and four were rated as good (Supplementary Table 4.1). Four studies did not meet the requirement for assessor blinding (4, 38, 54, 55), and three did not clearly report whether allocation concealment was implemented (38, 54, 55). Two studies failed to ensure therapist blinding (38, 54). Additionally, one study did not achieve completeness of primary outcome assessment (52), and another did not include dropout data in the final analysis (38).

According to the RoB 2 assessment (Supplementary Table 4.2), three studies were judged to have a low risk of bias and three to have some concerns, while one study was rated as having a high risk of bias. Four studies exhibited some concerns in Domain 5 (bias in selection of the reported result) (4, 38, 54, 55). In addition, one study showed some concerns in Domain 1 (bias arising from the randomization process) (55), one study in Domain 2 (bias due to deviations from intended interventions) (54), and one study in Domain 3 (bias due to missing outcome data) (38), respectively.

**Figure 2:**
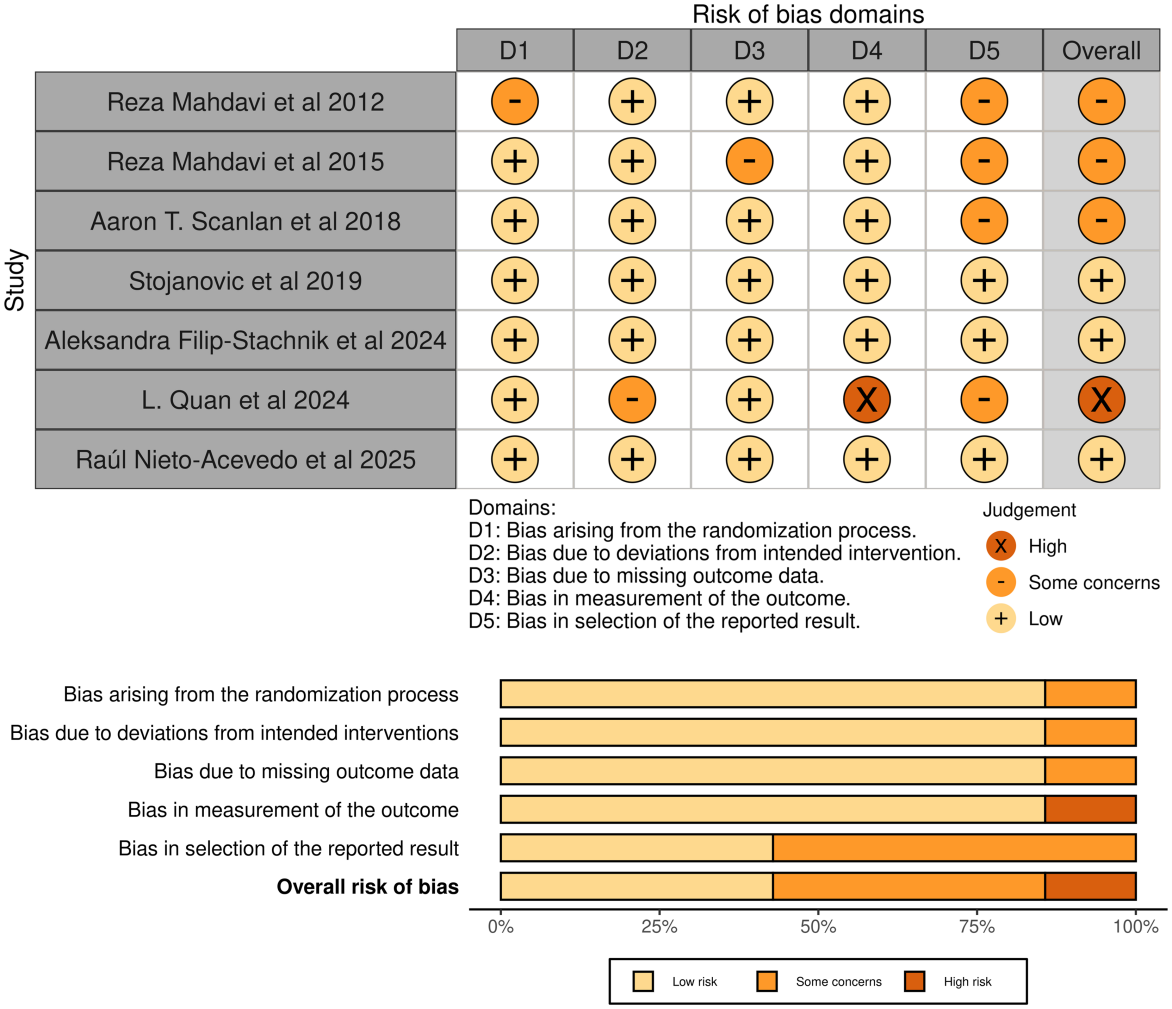
Risk of bias results.

Based on the PEDro and RoB 2 evaluations, a risk of bias plot was generated using the Robvis tool (56) (Figure 2). Sensitivity analyses excluding studies with concerns in RoB 2 Domain 5 are presented in (Supplementary Table 9). These concerns, particularly regarding selective reporting of results, were considered in the subsequent certainty of evidence assessment using the GRADE approach.

### 3.3 Participants and studies characteristics

The seven included studies collectively involved 101 female basketball players, with individual sample sizes ranging from 9 (52) to 26 (55). Participants were between 18 and 24 years of age. Except for two studies in which the training level was not specified (38, 55), the remaining studies reported that athletes had more than four years of systematic training experience. Except for one study that neither restricted nor reported habitual caffeine intake (52), all studies included athletes classified as low-to-moderate daily caffeine consumers.

Geographically, two studies were conducted in Australia, with one study conducted in each of Poland, Serbia, China, Iran, and Spain. A summary of study characteristics is presented in Table 1.

### 3.4 Caffeine supplementation protocol and dosage characteristics

The administered caffeine dose across the included studies ranged from 2.1 to 9 mg/kg. Six studies (2, 4, 38, 53–55) provided caffeine in capsule form, while one study (52) used caffeinated gum. Caffeine was administered 70 min before testing in two studies (38, 55), 60 min in four studies (2, 4, 53, 54), and 15 min in one study (52). All studies implemented a one-week washout period between experimental conditions. Regarding caffeine withdrawal, although specific procedures varied, most studies required participants to abstain from all dietary sources of caffeine for 48 h before testing. Detailed supplementation protocols and timing information are summarized in Table 1.

### 3.5 Menstrual cycle and oral contraceptives

Among the included studies, four did not report the menstrual cycle phase or its use in scheduling the experimental sessions (38, 52–54). Two studies conducted assessments during the luteal phase (2, 4), although neither specified the method used to determine menstrual cycle phase. One study performed testing on the 10th day following the onset of menstruation using a calendar-based approach (55). The specific menstrual cycle phase and its application in each study are summarized in Table 1. Furthermore, none of the seven included studies explicitly reported or controlled for oral contraceptive use.

### 3.6 Meta-analysis results

This review categorized *a priori* into physical performance and physiological outcomes based on their primary construct and measurement characteristics. Physical performance outcomes reflected externally observable, task-based measures of physical capacity (e.g., sprint speed, jump height, and power output), whereas physiological outcomes represented internally regulated perceptual, physiological, or biochemical responses (e.g., fatigue perception and physiological or biochemical markers). Caffeine-related adverse effects were also summarized. The forest plot illustrating the overall meta-analytic results is presented in Table 2, while the independent forest plots for each outcome measure are provided in Supplementary Figure 7. Following recommendations from previous studies, caffeine doses for the subgroup analyses were classified as ≤3 mg/kg (low), >3–6 mg/kg (moderate), and >6–≥9 mg/kg (high) (57–59). The results of the subgroup analyses are available in Supplementary Table 8.

Subgroup analyses were conducted on an exploratory basis to examine potential sources of heterogeneity (e.g., caffeine dose). Consistent with the Cochrane Handbook for Systematic Reviews of Interventions, these analyses should be interpreted with caution given the limited number of included studies and statistical power, and were not used to override the conclusions of the primary meta-analyses (60–62).

Leave-one-out sensitivity analyses (Supplementary Table 6) were conducted for outcomes including three or more studies. For jump, agility, and sprint performance, the direction of the pooled effects remained consistent after sequential removal of individual studies, indicating that the results were not driven by any single study. For fatigue-related outcomes, exclusion of Stojanovic et al. (2) resulted in a slight change in effect direction. Regarding anaerobic performance, omission of Mahdav et al. (38) led to a more pronounced change in effect direction, whereas exclusion of Stojanovic et al. (2) had only a minor impact. Additionally, for jump performance, the pooled effect became statistically significant after exclusion of Nieto-Acevedo et al. (53), for all other outcomes, statistical significance remained unchanged (*p* > 0.05).

In Sections 3.6.1–3.6.3, the combined sample size refers to the total number of participant-observations contributing to each meta-analysis outcome. When a single study reported multiple eligible outcomes or testing conditions within the same domain, these were included as separate effect sizes for the purposes of synthesis. Accordingly, the number of effect sizes may exceed the number of studies, and combined sample sizes may differ across outcomes.

#### 3.6.1 Sport-specific skill performance

##### 3.6.1.1 Shot accuracy

Two studies (52, 53) (five effect sizes; combined sample size = 108) were included in this analysis. The pooled meta-analysis did not demonstrate a statistically significant effect of caffeine on shooting accuracy in female basketball players (SMD = 0.36; 95% CI: −0.41 to 1.14; *p* = 0.263 > 0.05). Between-study heterogeneity was low (I^2^ = 18.6%, *p* = 0.263).

##### 3.6.1.2 Dribbling speed

Two studies (4, 52) (three effect sizes; combined sample size = 106) were included in this analysis. Because dribbling sprint speed was assessed by the time required to complete a predefined distance, negative effect sizes indicate performance improvement. The pooled meta-analysis did not demonstrate a statistically significant effect of caffeine on dribbling sprint speed in female basketball players (SMD: −0.10; 95% CI: −0.60 to 0.40; *p* = 0.616 > 0.05). Between-study heterogeneity was low (I^2^ = 0, *p* = 0.616).

#### 3.6.2 Physical performance

##### 3.6.2.1 Jump performance

Four studies (2, 52–54) (seven effect sizes; combined sample size = 234) assessed jump performance exclusively using vertical jump tests, including the countermovement jump (CMJ), squat jump, and basketball-specific technical jumps such as the Abalakov jump. The meta-analysis did not reveal a statistically significant effect of caffeine on jump performance in female basketball players (SMD = 0.63; 95% CI: −0.09 to 1.35; *p* = 0.080 > 0.05). Considerable between-study heterogeneity was observed (I^2^ = 77.2%, *p* = 0.08). Exploratory subgroup analyses suggested that jump type may moderate the effect of caffeine on jump performance (*p* < 0.01). Specifically, basketball-specific technical jumps (e.g., the Abalakov jump) demonstrated markedly larger effect sizes compared with coordination-based jumps and simple lower-limb–dominant jumps (Hedges’ g = 3.65 vs. 0.57 vs. 0.30). However, these findings should be interpreted with caution given the lack of a statistically significant overall effect and the high between study heterogeneity.

##### 3.6.2.2 Agility performance

Four studies (2, 52–54) (five effect sizes; combined sample size = 302) were included in this analysis. Because all agility outcomes were assessed using the time required to complete the task, negative effect sizes indicate improved performance. The pooled meta-analysis did not demonstrate a statistically significant effect of caffeine on agility performance in female basketball players (SMD = −0.24; 95% CI: −0.82 to 0.33; *p* = 0.376 > 0.05). Between-study heterogeneity was moderate (I^2^ = 54.9%, *p* = 0.376). Subgroup analyses suggested that caffeine dose may serve as a significant moderator, whereas measurement units, cognitive load, caffeine form, and task openness did not demonstrate significant moderating effects.

##### 3.6.2.3 Off-ball sprint speed

Three studies (2, 52, 53) were included in this analysis (three effect sizes; total sample size = 138). Based on the fixed distance sprint time evaluation, negative effect sizes indicate improvements in performance. The meta-analysis did not show a statistically significant effect of caffeine on off-ball sprint speed in female basketball players (SMD = −0.55; 95% CI: −1.11 to 0.00; *p* = 0.051 > 0.05), with low between-study heterogeneity (I^2^ = 0%, *p* = 0.051).

##### 3.6.2.4 Power output

Two studies (38, 54) (five effect sizes; combined sample size = 624) were included in this analysis. The pooled meta-analysis emonstrated a statistically significant moderate effect of caffeine on power output in female basketball players (SMD = 0.57; 95% CI: 0.09 to 1.04; *p* = 0.022 < 0.05), with moderate between-study heterogeneity (I^2^ = 40%, *p* = 0.022). Subgroup analyses showed a statistically significant difference across caffeine dosage (*p* < 0.01), whereas no significant subgroup differences were observed for outcome metric types (*p* = 0.18) or test modality or training level (*p* = 0.38).

##### 3.6.2.5 Anaerobic performance

Three studies (2, 38, 54) (eight effect sizes; combined sample size = 368) were included in this analysis. The suicide-run, a repeated shuttle sprint task reflecting anaerobic capacity demands (2, 9), was assessed using completion time, therefore effect sizes were expressed in the negative direction. Regarding blood lactate concentration, Quan et al. (54) measured it immediately after exercise, while Mahdavi et al. (38) measured it 5 min post-exercise. The pooled meta-analysis did not demonstrate a statistically significant effect of caffeine on anaerobic performance in female basketball players (SMD = −0.04; 95% CI: −0.80 to 0.71; *p* = 0.896 > 0.05). Between-study heterogeneity was high (I^2^ = 86.6%, *p* = 0.896). Subgroup analyses identified the predominant energy system targeted by the test may be a significant moderator, whereas caffeine dose, exercise modality, measurement method, and effect direction did not show significant moderating effects.

#### 3.6.3 Physiological function performance

##### 3.6.3.1 Fatigue-perception performance

Four studies (38, 52, 54, 55) (three effect sizes; combined sample size = 226) were included in this analysis. The pooled meta-analysis did not demonstrate a statistically significant effect of caffeine on overall fatigue perception in female basketball players (SMD = 0.12; 95% CI: −0.32 to 0.56; *p* = 0.55 > 0.05), with low between-study heterogeneity (I2 = 10%, *p* = 0.55).

##### 3.6.3.2 Physiological and biochemical markers

Two studies (54, 55) (seven effect sizes; combined sample size = 432) were included in this analysis. The pooled meta-analysis did not demonstrate a statistically significant effect of caffeine on overall physiological and biochemical markers in female basketball players (SMD = 0.53; 95% CI: −0.01 to 1.07; *p* = 0.052 > 0.05). Between-study heterogeneity was moderate (I^2^ = 65.8%, *p* = 0.052). Subgroup analyses indicated that effect direction may be an important moderator (*p* = 0.01), whereas marker type, sampling method, and caffeine dose did not show significant moderating effects.

#### 3.6.4 Potential adverse effects

Four studies (2, 4, 52, 53) reported adverse events within 24 h of caffeine ingestion (Table 2). The most frequent were tachycardia, headache, insomnia, and increased energy/activity. Filip-Stachnik (52) and Scanlan et al. (4) each reported a single adverse event: excessive sweating and an increased dribbling-error rate during skill execution.

**Table 2:** Tabular presentation of the overall meta-analysis forest plot results.

	No. of studies	CAF (*N*)	PLA (*N*)	SMD (95% CI)	*p* value	I^2^ (%)	GRADE
Sport-specific skill performance							
Shot accuracy	2	21	21	0.36 (−0.41, 1.14)	0.263	18.6	⨁⨁◯◯
Dribbling speed	2	19	19	−0.10 (−0.60, 0.40)	0.616	0	⨁⨁◯◯
Physical performance							
Jump performance	4	41	41	0.63 (−0.09, 1.35)	0.08	77.2	⨁◯◯◯
Agility performance	4	41	41	−0.24 (−0.82, 0.33)	0.376	54.9	⨁◯◯◯
Off-ball sprint speed	3	31	31	−0.55 (−1.11, 0.00)	0.051	0	⨁⨁◯◯
Power output	2	34	34	0.57 (0.09, 1.04)	0.022	40	⨁⨁◯◯
Anaerobic power	3	44	44	−0.04 (−0.80, 0.71)	0.896	86.6	⨁◯◯◯
Physiological function performance							
Fatigue-perception performance	4	53	53	0.12 (−0.32, 0.56)	0.55	10	⨁⨁◯◯
Physiological and biochemical markers	2	36	36	0.53 (−0.01, 1.07)	0.052	65.8	⨁◯◯◯

Values are presented as standardized mean differences (SMD) with 95% confidence intervals (CI) using random-effects models. Sample sizes (CAF and PLA) represent the total number of unique participants across included studies for each outcome; multiple effect sizes derived from the same study were counted only once to avoid double counting. Negative SMD values indicate improved performance when lower values reflect better outcomes (e.g., time-based measures).

Nieto-Acevedo et al. (53) reported significantly higher incidences of increased energy (33.0% vs. 7.0%) and tachycardia (22.0% vs. 7.0%) in the caffeine group compared with placebo (both *p* < 0.05). Stojanovic et al. (2) likewise observed higher adverse-event rates in the caffeine group (30% vs. 0%), although the differences were not statistically significant (*p* > 0.05) (see Table 3).

**Table 3:** Summary of caffeine-related adverse effects.

ID	First author	Side effects	Number of participants	CAF (%)	PLA (%)
1	Filip-Stachnik et al. (2022) (52)	Excessive sweating	9	11	0
2	Stojanovic et al. (2019) (2)	Headache	10	10	20
		Abdominal discomfort	10	20	10
		Muscle soreness	10	10	0
		Increased vigor/activeness	10	30	0
		Tachycardia	10	30	10
		Insomnia	10	10	20
		Increased urine output	10	10	10
		Increased anxiety	10	0	0
3	Scanlan et al. (4)	Dribble deficit (negative)	10	10	0
4	Nieto-Acevedo et al. (2025) (53)	Insomnia	12	11	13
		Tachycardia	12	22	7
		Anxiety	12	0	0
		Abdominal discomfort	12	0	0
		Headache	12	11	0
		Activeness	12	33	7
		Urine output	12	0	20

## 4 Discussion

### 4.1 Evidence summary

This systematic review synthesized evidence from seven randomized crossover trials involving a total of 101 individual female basketball players across all included studies. It aimed to evaluate the acute effects of caffeine on sport-specific skills, physical performance, and physiological and biochemical outcomes, and to examine the moderating roles of variables such as caffeine dose and test duration. The meta-analysis indicated that no statistically significant differences were observed across any of the analyzed performance variables.

In addition, the overall low certainty of evidence was partly driven by concerns related to selective reporting bias. According to the RoB 2 assessment, several included studies (4, 38, 54, 55) were judged as having some concerns in Domain 5 (bias in selection of the reported result), indicating insufficient transparency regarding pre-specified outcomes or analysis plans. This raises the possibility that favorable or statistically significant findings were preferentially reported, while null or unfavorable results may have been omitted. Such selective reporting can lead to an overestimation of intervention effects in meta-analyses. In line with GRADE guidance, this limitation contributed to downgrading the certainty of evidence for risk of bias, and the pooled estimates should therefore be interpreted with caution.

### 4.2 Acute effects of caffeine on specific basketball performance in female basketball players

In the present review, the meta-analysis did not demonstrate a statistically significant effect of caffeine on shooting accuracy among female basketball players. While Liu et al. (63) reported improvements in shooting accuracy following caffeine ingestion, several other studies have failed to observe meaningful effects of caffeine on accuracy-related outcomes (14, 24, 28, 30, 64, 65). These inconsistencies may be attributable to several confounding factors, such as baseline shooting proficiency or testing conditions, which are difficult to standardize across studies. Although caffeine has been suggested to influence alertness, cognitive processing, and hand–eye coordination during high-intensity exercise (66–68), the relevance of these mechanisms for shooting accuracy outcomes remains unclear. In addition, estrogen-mediated physiological differences lead to slower caffeine metabolism in women, potentially prolonging its beneficial effects on neuromuscular coordination and attenuating fatigue-induced declines in accuracy (69, 70). Such mechanisms may confer a physiological advantage for female athletes when performing precision-dependent tasks. Comparable caffeine-induced improvements in accuracy have been observed in other sports, including rugby passing, volleyball attacking, and serving accuracy in female table tennis players (19, 71, 72). However, most existing studies have not systematically examined sex-specific differences in shooting accuracy, and it remains unclear whether caffeine elicits distinct performance effects in male versus female athletes. Nevertheless, accuracy is a complex performance skill shaped by the interplay of multiple factors, including postural control, motor patterns, sensory feedback, technical proficiency, psychological regulation, and resistance to fatigue (73–75) which may further obscure the manifestation of caffeine-induced enhancements.

The present meta-analysis did not identify a statistically significant effect of caffeine on dribbling sprint speed in female basketball players, a finding consistent with studies involving mixed-sex and female team-sport athletes (28, 64). Dribbling is a complex basketball skill requiring multidimensional coordination, including direction-al changes, acceleration and deceleration, and continuous adaptation to dynamic environments (76). However, most existing studies have assessed dribbling using linear sprint tests, which have limited ecological validity compared with real game situations. Future research should incorporate more dynamic and game relevant assessment protocols, such as change of direction tasks, combined start stop actions, and tests integrating technical skill transitions, to more accurately evaluate the actual effects of caffeine on dribbling performance.

Both dribbling and shooting are precision-dependent basketball skills rooted in the technical proficiency acquired through long-term training. Consequently, caffeine is unlikely to directly enhance these sport-specific technical abilities. However, when athletes already possess well-developed technical and tactical foundations, appropriate caffeine supplementation may provide a meaningful marginal benefit to overall performance.

### 4.3 Acute effects of caffeine on physical performance in female basketball players

The meta-analysis in this review showed that the effect of caffeine on jump height in female basketball players did not reach statistical significance. Although the confidence interval included zero, indicating that the current evidence is insufficient to determine the true effect of caffeine on jump height in female basketball players, the point estimate of the pooled effect size demonstrated a moderate positive magnitude. This positive direction is consistent with the direction of effects reported in previous systematic reviews predominantly involving male or mixed-sex samples (24, 28, 73). The extremely large effect size observed for the Abalakov jump in the subgroup analysis should be interpreted with caution, as it is based on a single study with exceptionally low variability and may be associated with task- and measurement-specific characteristics, such as the inclusion of an approach phase and greater reliance on stretch–shortening cycle utilization. The absolute improvement was modest (approximately 2.3 cm), indicating that the large, standardized effect primarily reflects statistical scaling rather than a markedly enhanced physiological response to caffeine. Therefore, it should not be inferred that caffeine provides a greater ergogenic benefit for complex jumping tasks compared with simpler jump movements.

It is worth noting that some studies have shown that women exhibit a greater proportional area of type I muscle fibers in certain skeletal muscles compared with men (77, 78), and that type I muscle fibers demonstrate higher sensitivity to caffeine under *in vitro* conditions (79). Compared with male athletes, this muscle fiber characteristic may theoretically cause female basketball players to display different physiological responses to caffeine and may even confer a potential physiological advantage in basketball performance, where explosive jumping actions (e.g., rebounding) are critical. In addition, previous studies (26, 80, 81) have indicated that vertical jump performance typically reaches its lowest level in the early morning (most commonly between 08:00 and 12:00) and peaks in the late afternoon or early evening (most commonly between 16:00 and 20:00). Furthermore, three studies reported that a caffeine dose of 3 mg/kg significantly improved vertical jump height in both male and female basketball players in the morning (primarily around 09:00) (26), while also producing small-to-moderate improvements in the evening (19:00–21:00) (2, 24). This effect may be related to caffeine’s ability to enhance motor unit recruitment and increase muscle activation levels (22, 82), thereby improving muscle contractile properties and attenuating diurnal fluctuations in neuromuscular performance. Based on the above considerations, the authors speculate that caffeine ingestion in female basketball players may help maximize training adaptations across different times of day and provide marginal performance benefits during evening competition periods.

In our study, no statistically significant effect of caffeine on agility performance was observed in female basketball players. However, subgroup analyses suggested that caffeine dosage may represent an important moderating factor influencing agility performance. Interestingly, findings from the study by Quan et al. (54) suggest that the effect of caffeine on agility performance in female basketball players may follow an inverted U-shaped dose–response relationship. Specifically, during prolonged exercise, performance following the ingestion of 6 mg·kg^−1^ caffeine was superior to that observed with both lower (3 mg·kg^−1^) and higher (9 mg/kg) doses. This pattern may be partly attributable to the relatively limited ergogenic effects of lower doses (e.g., 3 mg/kg), as well as to an increased incidence of adverse effects at higher doses (e.g., 9 mg/kg), which could attenuate the beneficial influence of caffeine on agility-related performance (83). Notably, such a dose-dependent profile aligns with previous findings (57, 84) reported in female or mixed-sex athletic populations across other sport disciplines, in which moderate caffeine doses (e.g., 6 mg/kg) have been shown to confer greater improvements in agility or related performance outcomes, although such evidence has not yet been established in female basketball players. Meanwhile, agility performance itself exhibits a clear circadian rhythm, being typically lower in the morning (most commonly between 07:00 and 9:30) and reaching peak values in the late afternoon to evening (most commonly between 17:00 and 20:00) (80, 85, 86). Further research by Bougrine et al. (87) demonstrated that caffeine ingestion of 3 to 6 mg/kg at 08:00 effectively enhanced agility performance in female athletes, with a greater improvement observed at 6 mg·kg^−1^, which is consistent with an inverted U-shaped dose–response relationship, whereas caffeine intake during the evening (2, 24, 26) resulted in minimal performance benefits.

This meta-analysis did not identify a statistically significant effect of caffeine on maximal sprint performance in female basketball players. However, one study (52) reported that a caffeine dose of 2.3–3 mg/kg produced a moderate improvement in sprint performance in female basketball players, suggesting that very low doses of caffeine (<3 mg/kg) may have meaningful ergogenic potential. Two studies (2, 26) examining the effects of a low caffeine dose (3 mg/kg) demonstrated significant improvements in 10 m (ES = −0.63; −4.1%) and 20 m (ES = −0.41; −2.8%) sprint performance in female basketball players, with these effects being independent of circadian rhythm variations; no comparable improvements were observed in male basketball players at this dos. In contrast, male basketball players required a higher dose of 6 mg/kg to elicit a significant improvement in 20 m sprint performance (25). These findings suggest a potential sex-specific effect of caffeine on off-ball sprint performance, whereby female basketball players may obtain meaningful benefits at lower doses, whereas male players appear to require higher doses to achieve comparable ergogenic effects. However, it should be clearly noted that whether this effect is influenced by training status remains uncertain, and thus these conclusions should be interpreted with caution.

The present meta-analysis showed that caffeine ingestion may exert a moderate and statistically significant effect on power output in female basketball players (*p* < 0.05). Subgroup analyses indicated that caffeine dosage may moderate this effect (between-subgroup difference *p* < 0.01), with moderate doses (>3–6 mg/kg) appearing to elicit greater improvements. In contrast, outcome measures, testing modalities, and training status did not significantly moderate the observed effects (all between-subgroup differences *p* > 0.05), although significant pooled effects were observed across multiple outcome measures. Notably, Quan et al. (54) reported that a moderate caffeine dose (6 mg/kg) resulted in significantly greater improvements in power output compared with both a lower dose (3 mg/kg) and a higher dose (9 mg/kg) in female basketball players, a finding consistent with previous studies (88, 89) in female team-sport athletes and female soccer players. In contrast, Mahdavi et al. (38) reported no significant effect of a moderate caffeine dose (5 mg/kg) on power output in female basketball players, which aligns with earlier studies (90, 91) showing no significant improvement in power output during the Wingate anaerobic test following caffeine ingestion at approximately 6 mg/kg. These discrepant findings may be closely related to differences in power assessment methods. Studies reporting positive effects of caffeine on power output, such as those by Quan et al., predominantly employed field-based performance tests (e.g., repeated sprint tests), in which power variables are typically derived from sprint performance. In contrast, studies reporting null effects, such as that by Mah et al., primarily used cycle ergometer–based assessments (e.g., the Wingate test) to directly measure mechanical power output. Taken together, the effects of caffeine on power output in female basketball players remain inconclusive and appear to depend on both caffeine dose and the method used to assess power performance. Future studies using standardized power assessment protocols are warranted to clarify the dose–response relationship between caffeine supplementation and power output in this population.

In the present meta-analysis, no statistically significant ergogenic effect of caffeine on anaerobic performance was observed in female basketball players. In addition, the change in effect direction observed in the sensitivity analysis likely reflects the limited number of remaining effect sizes and the influence of individual studies, rather than a definitive reversal of the effect. Subgroup analyses indicated that, based on the current evidence, the predominant energy system required by the testing task may represent an important moderating factor influencing the effects of caffeine on anaerobic performance. It should be noted that Mahdavi et al. (38) reported that caffeine ingestion at a dose of 5 mg/kg significantly reduced the change in blood lactate concentration (SMD _diff_ = −2.25) as well as the magnitude of lactate accumulation (SMD _diff_ = −0.53). In contrast, the study by Quan et al. (54) demonstrated that caffeine ingestion at different doses (3, 6, and 9 mg/kg) exerted no significant effects on overall blood lactate concentrations, nor on the magnitude or direction of post-exercise lactate responses in female basketball players. These discrepancies may be attributable to methodological differences between studies, including variations in exercise protocols (Wingate test vs. high-intensity intermittent exercise) and the timing of blood lactate sampling (5 min post-exercise vs. immediately after exercise). Moreover, the ecological validity of using cycling-based power tests to assess basketball-specific performance, which involves more complex physiological and neuromuscular demands during training and competition, remains questionable.

### 4.4 Acute effects of caffeine on physiological function performance in female basketball players

The results of the meta-analysis indicated that caffeine ingestion did not produce a statistically significant effect on perceived fatigue (RPE) in female basketball players. Among the studies included in the present analysis, four investigations (2, 38, 52, 54) consistently reported that, across a dosage range of 2.1–9 mg/kg, caffeine exerted no significant effect or only a trivial effect on RPE in female basketball players. These findings are largely consistent with the study by Bougrine et al. (57) which demonstrated that caffeine ingestion at doses of 3 mg/kg and 6 mg/kg did not significantly affect RPE in female team-sport athletes, regardless of whether it was administered in the morning or in the evening. However, it should be noted that Stojanović et al. (26) reported that 3 mg/kg of caffeine significantly reduced RPE in male basketball players when ingested in the morning (approximately 10:00 h), a finding that contrasts with the results observed in female athletes. This discrepancy suggests that the time-of-day effects of caffeine on RPE may differ between sexes, and that evidence supporting a fatigue-attenuating effect of caffeine in female basketball players remains inconsistent and inconclusive. Although the present findings indicate that caffeine ingestion does not exert a significant overall effect on perceived fatigue in female basketball players, its potential underlying mechanisms remain worthy of consideration. Previous research (18) suggests that caffeine may attenuate fatigue and enhance muscular endurance during high-intensity exercise through mechanisms such as antagonism of adenosine receptors, increased central nervous system excitability, and modulation of neuromuscular regulation, at least under certain conditions. However, fatigue is a multifactorial and task-dependent phenomenon, and the physiological demands imposed by exercise duration and intensity may diminish or even obscure these potential ergogenic effects. Furthermore, given the inherently subjective nature of fatigue-related perceptual measures, the ecological validity of such indicators in basketball-specific performance research should be interpreted with caution.

Similarly, the present study did not identify any statistically significant effects of caffeine intake on the physiological or biochemical indices of female basketball players. Notably, previous studies (92–94) have suggested that caffeine ingestion, typically within the range of 3 to 7 mg/kg, may enhance the efficiency of energy substrate utilization, such as by increasing fat oxidation and reducing glycogen utilization. Additionally, caffeine may partially optimize physiological regulatory processes related to exercise performance, including elevations in plasma free fatty acids and epinephrine levels. In conjunction with the findings from the two studies (38, 55) included in the present analysis, namely, that ingestion of 5 mg/kg caffeine promoted post-exercise lactate clearance in female basketball players without exacerbating oxidative stress following high-intensity exercise, these results suggest that caffeine may confer beneficial and safe physiological advantages for female basketball players. However, it should be clearly noted that, given the absence of statistically significant effects of caffeine on physiological or biochemical indices observed in the present study, and considering that direct evidence in female basketball players remains limited, whether these potential physiological effects can be translated into meaningful improvements in exercise performance in this population still requires confirmation through future high-quality and sport-specific research.

**Figure 3:**
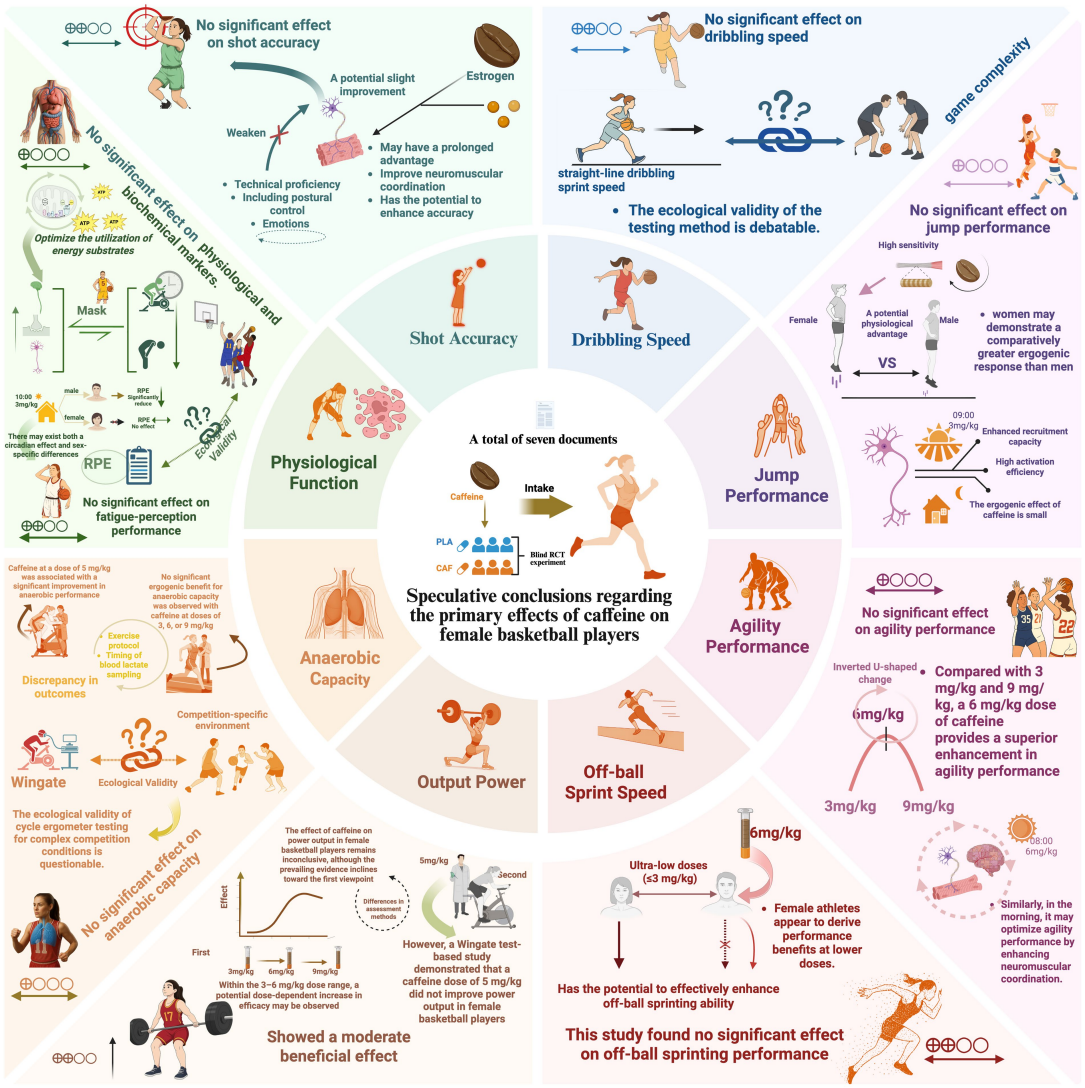
Conceptual framework. The observed outcomes did not show statistically significant effects in this study. Therefore, these findings should be interpreted with caution. The related discussions are informed by existing literature and remain speculative.

In this review, the primary findings discussed above are synthesized in the conceptual framework shown in Figure 3.

### 4.5 Caffeine dosage, timing, and form of intake

In the present review, one study (52) employed an ultra-low dose of caffeine (2.3 ± 0.2 mg/kg) administered in the form of caffeinated chewing gum to examine its effects on competitive performance in female basketball players. The results indicated small-to-moderate effect size improvements in sprint performance, agility, jump height, and free-throw accuracy; however, none of these changes reached statistical significance. Similarly, another study (95) using 3 mg/kg of caffeine chewing gum in male basketball players reported moderate effect size improvements in agility and jump performance, yet without statistically significant differences. Although the ergogenic dose of caffeine is commonly defined as 3–6 mg/kg (83), accumulating evidence (4, 73, 96, 97) suggests that very low doses (<3 mg/kg) may still elicit positive effects in certain sporting contexts. Taken together with findings from previous literature, the consistent trends toward performance enhancement observed under small sample size conditions, despite the absence of statistical significance, suggest that ultra-low-dose caffeine supplementation may exert potential ergogenic effects in female basketball players and possibly other athletic populations. These findings warrant further investigation using larger sample sizes and more robust study designs. Furthermore, all studies included in this review employed caffeine doses below 9 mg/kg. The prevailing view holds that very high doses of caffeine (>9 mg/kg) do not confer additional performance benefits (94), largely due to an increased likelihood of adverse effects. Nevertheless, the specific effects of such supra-high doses on female basketball players or team-sport athletes remain unclear and require further investigation.

Regarding ingestion timing and form, six studies included in this review administered caffeine in capsule form 60 min prior to exercise to coincide with the typical plasma caffeine peak window (30–60 min) (98). In contrast, the study employing caffeinated chewing gum administered caffeine 15 min before exercise, which is consistent with its pharmacokinetic characteristics, as caffeine delivered via chewing gum is rapidly absorbed through the oral mucosa, reaching peak plasma concentrations within approximately 15 min (99–101). Although this rapid absorption may shorten the required pre-exercise ingestion window, current evidence does not support a clear performance advantage of caffeinated chewing gum over traditional caffeine ingestion methods.

Notably, genetic polymorphisms related to caffeine may represent a potential mechanism underlying inter-individual variability in responses to caffeine supplementation (102–105), particularly involving genes associated with caffeine metabolism (e.g., CYP1A2) and adenosine receptor-mediated signaling pathways (e.g., ADORA2A). Although the relevance of these genetic factors has not been extensively studied in female basketball players, this area warrants further investigation. Future research should incorporate genetic analyses to determine whether specific genotypes are associated with differential ergogenic responses to caffeine in female basketball players.

### 4.6 Training status and menstrual cycle

Numerous studies investigating caffeine use in female athletes have suggested that training status and the menstrual cycle may modulate the ergogenic effects of caffeine (4, 24, 106–109). Among the studies included in the present review, only a subset controlled for the menstrual cycle (2, 4, 55), whereas participants were predominantly semi-professional and elite athletes. This methodological heterogeneity may, to some extent, account for the observed variability across study findings.

Available evidence indicates that athletes with higher training status tend to perform closer to the physiological and neuromuscular ceiling (110, 111), such that the ergogenic effects of caffeine often manifest as small effect sizes or marked inter individual variability (4, 24, 52, 106). In this context, if the menstrual cycle is not controlled, phase dependent hormonal influences on neuromuscular function (112) and energy substrate utilization (113) may further mask or attenuate the potential effects of caffeine. In contrast, among female athletes with lower training status, caffeine is more likely to elicit moderate or pronounced ergogenic effects, and these outcomes may be relatively less sensitive to menstrual cycle related fluctuations.

It is noteworthy that the menstrual cycle primarily consists of the follicular and luteal phases, which are characterized by fluctuations in estrogen and progesterone levels and may substantially influence physiological responses to exercise in female athletes (114, 115). Previous evidence (116–119) suggests that during the luteal phase, female athletes exhibit increased high-intensity running frequency, sprint count, and high-intensity running distance, along with enhanced aerobic capacity, whereas these performance indices show a statistically significant decline during the follicular phase. In contrast, shooting and rebounding performance appear to be superior during the follicular phase (120). Consequently, the ergogenic effects of caffeine supplementation may vary across menstrual cycle phases, and potential benefits could be attenuated or obscured by phase-dependent performance fluctuations.

From a mechanistic perspective, caffeine has been shown to increase intracellular Ca^2+^ and K^+^ availability. Enhanced Ca^2+^ release from the sarcoplasmic reticulum facilitates cross-bridge formation and contractile force production, whereas elevated K^+^ levels may stimulate Na^+^/K^+^-ATPase activity, thereby improving membrane excitability and delaying fatigue (121–123). In addition, estrogen and progesterone have been reported to exert opposite effects on muscle strength, with estrogen being positively associated and progesterone negatively associated with force production (119, 124, 125). Accordingly, female athletes in the follicular phase, characterized by elevated estrogen and relatively low progesterone concentrations, may exhibit greater strength and power, potentially amplifying the ergogenic effects of caffeine (126). Conversely, during the luteal phase, increased progesterone levels may attenuate neuromuscular performance and partially counteract or mask caffeine-induced benefits. Although some studies have reported no significant effect of menstrual cycle phase on strength performance, these hormonally mediated interactions remain a plausible regulatory mechanism and warrant further investigation.

Overall, training status may influence the magnitude of caffeine’s ergogenic effects, whereas menstrual cycle phase may affect their stability and direction. Therefore, without concurrent control or stratified analyses of these two factors, the true effects of caffeine on basketball performance in female athletes may be underestimated or appear inconsistent. This should be carefully considered when interpreting discrepancies across existing studies.

### 4.7 Potential adverse effects

Common potential adverse events include tachycardia, headache, insomnia, and increased energy/activity levels, which may be related to the hormonal regulation of caffeine metabolism. The luteal phase is characterized by elevated estrogen and progesterone levels (127), and a reduced caffeine clearance rate (128). Under this high estrogen environment, sustained adrenergic stimulation may increase susceptibility to caffeine-related adverse events (129). Furthermore, it is worth noting that Nieto-Acevedo et al. (53) reported a higher incidence of increased urinary output in the placebo group compared to the caffeine group (20% vs. 0%, *p* < 0.05), whereas Stojanovic et al. (2) did not observe significant changes. This finding contrasts with those in male or mixed-gender cohorts (26, 64), which may be related to exercise intensity: high-intensity exercise promotes the secretion of antidiuretic hormone (ADH, i.e., arginine vasopressin, AVP) (130), and estrogen lowers the threshold for AVP release (131).

Overall, the effects of caffeine on female basketball players are influenced by multiple factors. To enhance performance while minimizing adverse reactions, it is advisable to personalize caffeine dosage, with a recommendation to test caffeine use during training before official competitions to assess individual responses and tolerance.

### 4.8 Sensitivity analyses

In the leave-one-out sensitivity analyses (Supplementary Table 6), changes in effect direction were primarily observed for anaerobic power and fatigue-perception performance. Such directional shifts are more likely to reflect evidence fragility and task- or measurement-related heterogeneity rather than statistical calculation errors. Given the limited number of studies contributing to these outcomes (only three for anaerobic power and four for fatigue-perception performance), and the fact that the pooled effects in the primary analyses were close to null, even minor differences in individual study effects were sufficient to shift the pooled estimates across the null value, indicating limited stability of meta-analytic results based on a small evidence base.

Moreover, the primary analysis of anaerobic power demonstrated substantial heterogeneity (I^2^ = 86.6%), suggesting marked differences in how anaerobic capacity was defined and assessed across studies. For example, anaerobic outcomes derived from direct power output, composite performance tests, or metabolic response indices may not reflect the same underlying physiological construct. The subgroup analyses further support this interpretation, indicating that construct-level differences among anaerobic power measures amplified the influence of individual studies on the overall estimates. In contrast, fatigue-perception outcomes are inherently more subjective and therefore more susceptible to scale-related variability and methodological factors; the return of pooled effects toward null values after omitting individual studies further suggests that the evidence base for this outcome remains limited.

Overall, these directional changes indicate that, under conditions of a small number of included studies and heterogeneous outcome constructs, the available evidence for these outcomes remains fragile, and the corresponding conclusions should be interpreted with caution.

### 4.9 Study limitations

This systematic review has several limitations. First, only seven studies were eligible for inclusion, which limits the robustness of the pooled estimates and warrants caution when interpreting the findings in this specific population. While the focus on female basketball players is important, the current evidence base remains limited. Moreover, participant age and competitive level varied across studies, and although these characteristics were recorded when reported, potential age or expertise related differences could not be systematically examined and should be considered when interpreting the findings. Therefore, the present analysis should be regarded as a preliminary exploration of the potential performance effects of caffeine in female basketball players, and additional well-designed studies are needed to better understand and confirm these results. Second, substantial heterogeneity was observed in the performance assessment protocols, and some tests may have limited relevance to basketball-specific movements, potentially affecting ecological validity. Finally, basketball performance is shaped by interacting technical, physical, and tactical factors; thus, caffeine-related physiological effects represent only one component of performance and the present findings should not be overinterpreted.

Based on the available evidence, several avenues warrant further investigation: (1) the effects of caffeine on decision-making and physical function during critical phases of competition (e.g., end-game periods) or under acute fatigue; (2) interactions between hormonal fluctuations across the menstrual cycle and caffeine metabolism in female athletes, as well as the feasibility of adjusting caffeine intake strategies accordingly to sustain performance; (3) sex-specific mechanisms underlying caffeine responsive-ness, particularly how physiological differences shape individual response patterns; and (4) the potential impact of interactions between caffeine and genetic variability in female basketball players. These remain important yet underexplored areas for future research.

## 5 Conclusion

In summary, The results of this meta-analysis indicate that caffeine doses ranging from 2.1 to 9 mg/kg did not produce significant effects on a range of basketball-specific skill tests (i.e., shooting accuracy and dribbling sprint performance), physical performance measures (including jump height, agility, sprint speed without the ball, and anaerobic capacity), or physiological outcomes (such as perceived fatigue and physiological and biochemical markers) in female basketball players. However, caffeine ingestion demonstrated a moderate and statistically significant effect on power output, with subgroup analyses suggesting that moderate doses (>3–6 mg/kg) may elicit greater improvements. Caffeine ingestion may also be associated with the occurrence of common side effects, including insomnia, headache, and excessive sweating.

Overall, based on the available evidence, caffeine ingestion does not appear to consistently enhance sport-specific skills, physical performance, or physiological outcomes in female basketball players. It should be explicitly acknowledged that, given the limited number of eligible studies and the presence of methodological heterogeneity, the discussions and analyses of performance-related outcomes presented in this study should be regarded as a preliminary and exploratory assessment of the effects of caffeine in women’s basketball. Accordingly, the findings of this meta-analysis should be interpreted with caution. Accordingly, the findings of this meta-analysis should be interpreted with caution.

## 6 Practical applications

Based on the findings of the present meta-analysis, caffeine cannot currently be regarded as an effective supplementation strategy for enhancing performance in female basketball players and may be accompanied by potential adverse effects, such as tachycardia and insomnia. In applied practice, caffeine use should be informed by prior individualized testing and cautious planning of intake dosage, timing, and form, while considering hormonal fluctuations potentially associated with the menstrual cycle as well as the athlete’s training status.

Given that basketball games are commonly scheduled in the evening, pre competition caffeine ingestion may increase the risk of insomnia, thereby exerting a negative influence on post competition recovery. This issue is particularly pronounced in tournament-based competitions, in which adequate sleep and recovery between consecutive matches are critical for maintaining performance. In this context, it is recommended that any potential performance related benefits of caffeine be carefully weighed against the impact of its side effects on recovery. Individual responses to caffeine should be evaluated during regular training sessions rather than during competition, with emphasis placed on individualized caffeine supplementation strategies that account for inter individual variability among female basketball players.

## Data Availability

The original contributions presented in the study are included in the article/Supplementary material, further inquiries can be directed to the corresponding author.
