# Supplementary material for: A systematic review and meta-analysis of the evidence on the acute effects of caffeine on sport-specific skills, physical performance, and physiological function in female basketball players
Source: Front Nutr. 2026 Feb 23;13:1766993. doi: 10.3389/fnut.2026.1766993 (PMC12968231; doi:10.3389/fnut.2026.1766993)
Supplement: Supplementary file 1 [file Supplementary_file_1.docx]

Supplementary Material

| **Section/Topic** | **Item #** | **Checklist item** | **Section headings** |
| --- | --- | --- | --- |
| **A systematic review and meta-analysis of the evidence on the acute effects of caffeine on sport-specific skills, physical performance, and physiological function in female basketball players** | | |  |
| Title | 1 | Identify the report as a systematic review， meta-analysis， or both. | Title |
|  | | |  |
| Structured summary | 2 | Provide a structured summary including， as applicable: background; objectives; data sources; study eligibility criteria， participants， and interventions; study appraisal and synthesis methods; results; limitations; conclusions and implications of key findings; systematic review registration number. | Abstract |
| **INTRODUCTION** | | |  |
| Rationale | 3 | Describe the rationale for the review in the context of what is already known. | Background |
| Objectives | 4 | Provide an explicit statement of questions being addressed with reference to participants， interventions， comparisons， outcomes， and study design (PICOS). | Objectives |
| **METHODS** | | |  |
| Protocol and registration | 5 | Indicate if a review protocol exists， if and where it can be accessed (e.g.， Web address)， and， if available， provide registration information including registration number. | Search Strategy |
| Eligibility criteria | 6 | Specify study characteristics (e.g.， PICOS， length of follow-up) and report characteristics (e.g.， years considered， language， publication status) used as criteria for eligibility， giving rationale. | Eligibility Criteria |
| Information sources | 7 | Describe all information sources (e.g.， databases with dates of coverage， contact with study authors to identify additional studies) in the search and date last searched. | Search Strategy and Eligibility Criteria |
| Search | 8 | Present full electronic search strategy for at least one database， including any limits used， such that it could be repeated. | Supplementary Table 2 |
| Study selection | 9 | State the process for selecting studies (i.e.， screening， eligibility， included in systematic review， and， if applicable， included in the meta-analysis). | Study Selection |
| Data collection process | 10 | Describe method of data extraction from reports (e.g.， piloted forms， independently， in duplicate) and any processes for obtaining and confirming data from investigators. | Data Extraction and Transformation |
| Data items | 11 | List and define all variables for which data were sought (e.g.， PICOS， funding sources) and any assumptions and simplifications made. | Supplementary Table 3 |
| Risk of bias in individual studies | 12 | Describe methods used for assessing risk of bias of individual studies (including specification of whether this was done at the study or outcome level)， and how this information is to be used in any data synthesis. | Quality Assessment of Included Studies and Evidence Grading |
| Summary measures | 13 | State the principal summary measures (e.g.， risk ratio， difference in means). | Statistical Analysis |
| Synthesis of results | 14 | Describe the methods of handling data and combining results of studies， if done， including measures of consistency (e.g.， *I*^2^) for each meta-analysis. | Statistical Analysis |
| Risk of bias across studies | 15 | Specify any assessment of risk of bias that may affect the cumulative evidence (e.g.， publication bias， selective reporting within studies). | Quality Assessment of Included Studies |
| Additional analyses | 16 | Describe methods of additional analyses (e.g.， sensitivity or subgroup analyses， meta-regression) ， if done， indicating which were pre-specified. | Statistical Analysis |
| **RESULTS** | | |  |
| Study selection | 17 | Give numbers of studies screened， assessed for eligibility， and included in the review， with reasons for exclusions at each stage， ideally with a flow diagram. | Literature Screening Process and Figure 1 |
| Study characteristics | 18 | For each study， present characteristics for which data were extracted (e.g.， study size， PICOS， follow-up period) and provide the citations. | Literature Screening Process and Table 1 |
| Risk of bias within studies | 19 | Present data on risk of bias of each study and， if available， any outcome level assessment (see item 12). | Risk of Bias Assessment in Included Studies and Supplementary Table 4 |
| Results of individual studies | 20 | For all outcomes considered (benefits or harms)， present， for each study:(a) simple summary data for each intervention group (b) effect estimates and confidence intervals， ideally with a forest plot. | Meta-Analysis Results and Table 2; Supplementary Figure 7 |
| Synthesis of results | 21 | Present results of each meta-analysis done， including confidence intervals and measures of consistency. | Meta-Analysis Results and Table 2 |
| Risk of bias across studies | 22 | Present results of any assessment of risk of bias across studies (see Item 15). | Supplementary Table 4 |
| Additional analysis | 23 | Give results of additional analyses， if done (e.g.， sensitivity or subgroup analyses， meta-regression [see Item 16]). | Supplementary Table 6/8/9 |
| **DISCUSSION** | | |  |
| Summary of evidence | 24 | Summarize the main findings including the strength of evidence for each main outcome; consider their relevance to key groups (e.g.， healthcare providers， users， and policy makers). | Evidence Summary |
| Limitations | 25 | Discuss limitations at study and outcome level (e.g.， risk of bias)， and at review-level (e.g.， incomplete retrieval of identified research， reporting bias). | Limitations |
| Conclusions | 26 | Provide a general interpretation of the results in the context of other evidence， and implications for future research. | Conclusions |
| **FUNDING** | | |  |
| Funding | 27 | Describe sources of funding for the systematic review and other support (e.g.， supply of data); role of funders for the systematic review. | Funding |

# Supplementary Table 1. PRISMA 2020 Checklist

# Supplementary Table 2. Search Strategy

| **PubMed（982 article）：**  ((((((((((((((((((((caffeine[MeSH Terms]) OR (caffeine[Title/Abstract])) OR (1,3,7-trimethylxanthine[Title/Abstract])) OR (No Do[Title/Abstract])) OR (Caffedrine[Title/Abstract])) OR (Coffeinum N[Title/Abstract])) OR (Coffeinum Purrum[Title/Abstract])) OR (Dexitac[Title/Abstract])) OR (Durvitan[Title/Abstract])) OR (Percoffedrinol N[Title/Abstract])) OR (Vivarin[Title/Abstract])) OR (Percutaféine[Title/Abstract])) OR (Quick-Pep[Title/Abstract])) OR (QuickPep[Title/Abstract])) OR (Quick Pep[Title/Abstract])) OR (tea[Title/Abstract])) OR (coffee[Title/Abstract])) OR (energy drink[Title/Abstract])) OR (caffeinated[Title/Abstract])) AND (((basketball*[MeSH Terms]) OR (basketball*[Title/Abstract])) OR (netball*[Title/Abstract]))) AND ((((((((((((athlete*[MeSH Terms]) OR (athlete*[Title/Abstract])) OR (professional athlete*[Title/Abstract])) OR (athlete*, profession-al[Title/Abstract])) OR (elite athlete*[Title/Abstract])) OR (athlete*, elite[Title/Abstract])) OR (college athlete*[Title/Abstract])) OR (athlete*, college[Title/Abstract])) OR (player*[Title/Abstract])) OR (competitor*[Title/Abstract])) OR (sportsman[Title/Abstract])) OR (sportswoman[Title/Abstract])) |
| --- |
| **Web of Science（708 article）：**  caffeine (Topic) or caffeine (Title) or caffeine (Abstract) or 1,3,7-trimethylxanthine (Title) or 1,3,7-trimethylxanthine (Abstract) or No Do (Title) or No Do (Abstract) or Caffedrine (Title) or Caffedrine (Abstract) or Coffeinum N (Title) or Coffeinum N (Abstract) or Coffeinum Purrum (Title) or Coffeinum Purrum (Abstract) or Dexitac (Title) or Dexitac (Abstract) or Durvitan (Title) or Durvitan (Abstract) or Percoffedrinol N (Title) or Percoffedrinol N (Abstract) or Vivarin (Title) or Vivarin (Abstract) or Percutaféine (Title) or Percutaféine (Abstract) or Quick-Pep (Title) or Quick-Pep (Abstract) or QuickPep (Title) or QuickPep (Abstract) or Quick Pep (Title) or Quick Pep (Abstract) or tea (Title) or tea (Abstract) or coffee (Title) or coffee (Abstract) or energy drink (Title) or energy drink (Abstract) or caffeinated (Title) or caffeinated (Abstract) and Preprint Citation Index (Exclude – Database) And (((((TS=(basketball*)) OR TI=(basketball*)) OR AB=(basketball*)) OR TS=( netball*)) OR TI=( netball*)) OR AB=( netball*) and Preprint Citation Index (Exclude – Database) And ((((((((((((((((((((((TS=(athlete*)) OR TI=(athlete*)) OR AB=(athlete*)) OR TI=(professional athlete*)) OR AB=(professional athlete*)) OR TI=(athlete*, professional)) OR AB=(athlete*, professional)) OR TI=(elite athlete*)) OR AB=(elite athlete*)) OR TI=(athlete*, elite)) OR AB=(athlete*, elite)) OR TI=(college athlete*)) OR AB=(college athlete*)) OR TI=( athlete*, college)) OR AB=( athlete*, college)) OR TI=(player*)) OR AB=(player*)) OR TI=(competitor*)) OR AB=(competitor*)) OR TI=(sportsman)) OR AB=(sportsman)) OR TI=(sportswoman)) OR AB=(sportswoman) and Preprint Citation Index (Exclude – Database) |
| **EMBASE（236 article）：**  caffeine OR caffeine:ab,ti OR '1,3,7 trimethylxanthine' OR '1,3,7 trimethylxanthine':ab,ti OR (no AND do) OR 'no do':ab,ti OR caffedrine OR caffedrine:ab,ti OR (coffeinum AND n) OR 'coffeinum n':ab,ti OR (coffeinum AND purrum) OR 'coffeinum purrum':ab,ti OR dexitac OR dexitac:ab,ti OR durvitan OR durvitan:ab,ti OR (percoffedrinol AND n) OR 'percoffedrinol n':ab,ti OR vivarin OR vivarin:ab,ti OR percutaféine OR percutaféine:ab,ti OR 'quick pep' OR quickpep OR quickpep:ab,ti OR (quick AND pep) OR 'quick pep':ab,ti OR tea OR tea:ab,ti OR coffee OR coffee:ab,ti OR (energy AND drink) OR 'energy drink':ab,ti OR caffeinated OR caffeinated:ab,ti AND basketball* OR basketball*:ab,ti OR netball* OR netball*:ab,ti AND athlete* OR athlete*:ab,ti OR (professional AND athlete*) OR 'professional athlete*':ab,ti OR (athlete*, AND professional) OR 'athlete*, professional':ab,ti OR (elite AND athlete*) OR 'elite athlete*':ab,ti OR (athlete*, AND elite) OR 'athlete*, elite':ab,ti OR (college AND athlete*) OR 'college athlete*':ab,ti OR (athlete*, AND college) OR 'athlete*, college':ab,ti OR player* OR player*:ab,ti OR competitor* OR competitor*:ab,ti OR sportsman OR sportsman:ab,ti OR sportswoman OR sportswoman:ab,ti |
| **Cochrane（90 article）：**  #1(caffeine) OR (caffeine):ti,ab,kw OR (1,3,7 trimethylxanthine) OR (1,3,7 trimethylxanthine):ti,ab,kw;#2(No Do) OR (No Do):ti,ab,kw OR (Caffedrine) OR (Caffedrine):ti,ab,kw;#3(Coffeinum N) OR (Coffeinum N):ti,ab,kw OR (Coffeinum Purrum) OR (Coffeinum Purrum):ti,ab,kw;#4(Coffeinum N) OR (Coffeinum N):ti,ab,kw OR (Coffeinum Purrum) OR (Coffeinum Purrum):ti,ab,kw;#5(Dexitac) OR (Dexitac):ti,ab,kw OR (Durvitan) OR (Durvitan):ti,ab,kw;#6(Percoffedrinol N) OR (Percoffedrinol N):ti,ab,kw OR (Vivarin) OR (Vivarin):ti,ab,kw;#7(Percutaféine) OR (Percutaféine):ti,ab,kw OR (Quick-Pep) OR (Quick-Pep):ti,ab,kw;#8(QuickPep) OR (QuickPep):ti,ab,kw OR (Quick Pep) OR (Quick Pep):ti,ab,kw;#9(tea) OR (tea):ti,ab,kw OR (coffee) OR (coffee):ti,ab,kw;#10(energy drink) OR (energy drink):ti,ab,kw OR (caffeinated) OR (caffeinated):ti,ab,kw;#11（#1 OR #2 OR #3 OR #4 OR #5 OR #5 OR #6 OR #7 OR #8 OR #9 OR #10）;#12(basketball*) OR (basketball*):ti,ab,kw OR (netball*) OR (netball*):ti,ab,kw;#13(athlete*) OR (athlete*):ti,ab,kw OR (professional athlete*) OR (professional athlete*):ti,ab,kw;#14(athlete*, professional) OR (athlete*, professional):ti,ab,kw OR (elite athlete*) OR (elite athlete*):ti,ab,kw;#15(athlete*, elite) OR (athlete*, elite):ti,ab,kw OR (college athlete*) OR (college athlete*):ti,ab,kw;#16(athlete*, college) OR (athlete*, college):ti,ab,kw OR (player*) OR (player*):ti,ab,kw;#17(competitor*) OR (competitor*):ti,ab,kw OR (sportsman) OR (sportsman):ti,ab,kw;#18(sportswoman) OR (sportswoman):ti,ab,kw;#19（#13 OR #14 OR #15 OR #16 OR #17 OR #18）;#20(female*) OR (female*):ti,ab,kw OR (women*) OR (women*):ti,ab,kw;#21(girl*) OR (girl*):ti,ab,kw;#22（#20 OR #21） |
| **Scopus（260 article）:**  ( ( TITLE-ABS-KEY ( caffeine ) OR TITLE-ABS-KEY ( 1,3,7-trimethylxanthine ) OR TITLE-ABS-KEY ( no AND do ) OR TITLE-ABS-KEY ( caffedrine ) OR TITLE-ABS-KEY ( coffeinum AND n ) OR TITLE-ABS-KEY ( coffeinum AND purrum ) OR TITLE-ABS-KEY ( durvitan ) OR TITLE-ABS-KEY ( percoffedrinol AND n ) OR TITLE-ABS-KEY ( vivarin ) OR TITLE-ABS-KEY ( percutaféine ) OR TITLE-ABS-KEY ( quick-pep ) OR TITLE-ABS-KEY ( quickpep ) OR TITLE-ABS-KEY ( quick AND pep ) OR TITLE-ABS-KEY ( tea ) OR TITLE-ABS-KEY ( coffee ) OR TITLE-ABS-KEY ( energy AND drink ) OR TITLE-ABS-KEY ( caffeinated ) ) ) AND ( ( TITLE-ABS-KEY ( basketball* ) OR TITLE-ABS-KEY ( netball* ) ) ) AND ( ( TITLE-ABS-KEY ( athlete* ) OR TITLE-ABS-KEY ( professional AND athlete* ) OR TITLE-ABS-KEY ( athlete*, AND professional ) OR TITLE-ABS-KEY ( elite AND athlete* ) OR TITLE-ABS-KEY ( athlete*, AND elite ) OR TITLE-ABS-KEY ( college AND athlete* ) OR TITLE-ABS-KEY ( athlete*, AND college ) OR TITLE-ABS-KEY ( player* ) OR TITLE-ABS-KEY ( competitor* ) OR TITLE-ABS-KEY ( sportsman ) OR TITLE-ABS-KEY ( sportswoman ) ) |
| **ProQuest（153 article）**  S1: mainsubject(caffeine) OR title(caffeine) OR abstract(caffeine) OR title(1,3,7trimethylxanthine) OR abstract(1,3,7trimethylxanthine) OR title (No Do) OR abstract(No Do) OR title(Caffedrine) OR abstract(Caffedrine);S2 title(Coffeinum N) OR abstract(Coffeinum N) OR title(Coffeinum Purrum) OR abstract(Coffeinum Purrum) OR title(Dexitac) OR abstract(Dexitac) OR title(Durvitan) OR abstract(Durvitan) OR title(Percoffedrinol N) OR abstract(Percoffedrinol N);S3 title(Vivarin) OR abstract(Vivarin) OR title(Percutaféine) OR abstract(Percutaféine) OR title(Quick-Pep) OR abstract(Quick-Pep) OR title(QuickPep) OR abstract(QuickPep) OR title(Quick Pep) OR abstract(Quick Pep);S4 title(tea) OR abstract(tea) OR title(coffee) OR abstract(coffee) OR title(energy drink ) OR abstract(energy drink ) OR title(caffeinated) OR abstract(caffeinated);S5 [S1] OR [S2] OR [S3] OR [S4]; S6 mainsubject(athlete*) OR title(athlete*) OR abstract(athlete*) OR title(professional athlete*) OR abstract(professional athlete*) OR title(athlete*, professional) OR abstract(athlete*, professional) OR title(elite athlete*) OR abstract(elite athlete*);S7 title(athlete*, elite) OR abstract(athlete*, elite) OR title(college athlete*) OR abstract(college athlete*) OR title(athlete*, college) OR abstract(athlete*, college) OR title(player*) OR abstract(player*) OR title(competitor*) OR abstract(competitor*);S8 title(sportsman) OR abstract(sportsman) OR title(sportswoman) OR abstract(sportswoman);S9[S6] OR [S7] OR [S8];S10 mainsubject(basketball*) OR title(basketball*) OR abstract(basketball*) OR title(netball*) OR abstract(netball*);S11 [S5] AND [S9] AND [S10] |
| **EBSCOhost（111 article）**  S1 SU caffeine OR TI caffeine OR AB caffeine OR TI 1,3,7-trimethylxanthine OR AB 1,3,7-trimethylxanthine OR TI No Do OR AB No Do; S2 TI Caffedrine OR AB Caffedrine OR TI Coffeinum N OR AB Coffeinum N OR TI Coffeinum Purrum OR AB Coffeinum Purrum; S3 TI Dexitac OR AB Dexitac OR TI Durvitan OR AB Durvitan OR TI Percoffedrinol N OR AB Percoffedrinol N; S4 TI Vivarin OR AB Vivarin OR TI Percutaféine OR AB Percutaféine OR TI Quick-Pep OR AB Quick-Pep; S5 TI QuickPep OR AB QuickPep OR TI Quick Pep OR AB Quick Pep OR TI tea OR AB tea ; S6 TI coffee OR AB coffee OR TI energy drink OR AB energy drink OR TI caffeinated OR AB caffeinated; S7 S1 OR S2 OR S3 OR S4 OR S5 OR S6; S8 SU athlete* OR TI athlete* OR AB athlete* OR TI professional athlete* OR AB professional athlete* OR TI athlete*, professional OR AB athlete*, professional ; S9 TI elite athlete* OR AB elite athlete* OR TI athlete*, elite OR AB athlete*, elite OR TI college athlete* OR AB college athlete*; S10 TI athlete*, college OR AB athlete*, college OR TI player* OR AB player* OR TI competitor* OR AB competitor* ;S11 TI sportsman OR AB sportsman OR TI sportswoman OR AB sportswoman; S12 S8 OR S9 OR S10 OR S11; S13 SU basketball* OR TI basketball* OR AB basketball* OR TI netball* OR AB netball* ; S14 S7 AND S13 AND S12 |

# Supplementary Table 3. Studies eligibility criteria

|  | **Inclusion** | **Exclusion** |
| --- | --- | --- |
| **P** | Female basketball players (age ≥ 18 years) (no strict restrictions on training level). | Individuals with disabilities, wheelchair athletes, or subjects with long-term habituation to caffeine effects. |
| **I** | Pharmacokinetic Interventions of Caffeine | Studies that utilized mixed reagents with non-caffeine primary components for experimentation, or did not specify caffeine intake protocols, or failed to document nutritional intake substances. |
| **C** | Subjects who did not receive any drug intake or received drug intake that would not potentially affect the study outcomes and completed the same testing procedures as the experimental group under blind conditions. | Lacked a comparison group (i.e., single-group design |
| **O** | Shooting Accuracy: such as total score or shooting percentage under specified number of attempts  Dribbling Speed: Such as the time required to sprint a specified distance while dribbling  Jump Performance: Such as countermovement jump, jump height, squat jump, and jump height with or without arm swing.  Agility: Such as the Lane Agility Drill, Stroop Test, and T-test.  Speed: The time required for athletes to complete an all-out sprint over a specified distance without the ball.  Power output: Such as peak power output, mean power output, and player load  Anaerobic Power: Such as Blood lactate concentration or accumulation rate, athlete's power output during Wingate test, and suicide runs.  Fatigue: Such as fatigue index, RPE (Rating of Perceived Exertion), and perceptual responses performance  Physiological and Biochemical indicators: Such as malondialdehyde, antioxidant capacity, creatine kinase, white blood cells, lymphocyte, and granulocyte. | Studies with outcome measures that did not cover the effects of caffeine on physical performance. |
| **S** | Randomized control trails or control trails | Unpublished data or gray literature, and research employing meta-analyses or systematic reviews were excluded. |
| Note: P, participants; I, intervention; C, control; O, outcome; S, study design | | |

# Supplementary Table 4. Physiotherapy Evidence Database (PEDro) and RoB2 tool

## Supplementary Table 4.1. Physiotherapy Evidence Database (PEDro)

| **Study** | **The first author** | **Year of Publication** | **D1** | **D2** | **D3** | **D4** | **D5** | **D6** | **D7** | **D8** | **D9** | **D10** | **D11** | **Total** |  |
| --- | --- | --- | --- | --- | --- | --- | --- | --- | --- | --- | --- | --- | --- | --- | --- |
| 1 | Reza Mahdavi | 2012 | Y | 1 | 0 | 1 | 1 | 1 | 0 | 1 | 1 | 1 | 1 | 8 |  |
| 2 | Reza Mahdavi | 2015 | Y | 1 | 0 | 1 | 1 | 1 | 0 | 1 | 0 | 1 | 1 | 7 |  |
| 4 | Aaron T. Scanlan | 2019 | Y | 1 | 1 | 1 | 1 | 1 | 0 | 1 | 1 | 1 | 1 | 9 |  |
| 5 | Stojanovic | 2019 | Y | 1 | 1 | 1 | 1 | 0 | 1 | 1 | 1 | 1 | 1 | 10 |  |
| 6 | Aleksandra Filip-Stachnik | 2024 | Y | 1 | 1 | 1 | 1 | 1 | 1 | 0 | 1 | 1 | 1 | 8 |  |
| 7 | L. Quan | 2024 | Y | 1 | 0 | 1 | 1 | 0 | 0 | 1 | 1 | 1 | 1 | 7 |  |
| 8 | Raúl Nieto-Acevedo | 2025 | Y | 1 | 1 | 1 | 1 | 1 | 1 | 1 | 1 | 1 | 1 | 10 |  |
| **Table note:** “Y” or “1” indicates that the study meets the criterion; “N” or “0” indicates that the study does not meet the criterion. | | | | | | | | | | | | | | | |

## Supplementary Table 4.2. Risk bias assessment tool RoB2

| **Study** | **D1** | **D2** | **D3** | **D4** | **D5** | **Overall** |
| --- | --- | --- | --- | --- | --- | --- |
| Reza Mahdavi et al 2012 | Some concerns | Low | Low | Low | Some concerns | some concerns |
| Reza Mahdavi et al 2015 | Low | Low | Some concerns | Low | Some concerns | Some concerns |
| Aaron T. Scanlan et al 2019 | Low | Low | Low | Low | Some concerns | Some concerns |
| Stojanovic et al 2019 | Low | Low | Low | Low | Low | low |
| Aleksandra Filip-Stachnik et al 2024 | Low | Low | Low | Low | Low | low |
| L. Quan et al 2024 | Low | Some concerns | Low | High | Some concerns | High |
| Raúl Nieto-Acevedo et al 2025 | Low | Low | Low | Low | Low | low |

# Supplementary Table 5. GRADE-based evidence rating for the conclusions of this study

| **Outcome** | **No of participants (Studies)** | **Certainty Assessment** | | | | | **Standardized Mean**  **effect (95% CI) †** | **GRADE*** |
| --- | --- | --- | --- | --- | --- | --- | --- | --- |
|  |  | **Risk of Bias** | **Inconsistency** | **Indirectness** | **Imprecision** | **Other** |  |  |
| **Caffeine (CAF) *versus* Placebo (PLA)** | | | | | | | |  |
| **Shot accuracy** | **108 (2 RCT)** | **Sone serious** | **Not serious** | **Not serious** | **Some serious** | **None** | **0.36（-0.41 to 1.14）** | **⨁⨁◯◯ Low** |
| **Dribbling sprint speed** | **106 (2 RCT)** | **Sone serious** | **Not serious** | **Not serous** | **Some serious** | **None** | **-0.10（-0.6 to 0.40）** | **⨁⨁◯◯ Low** |
| **Jump Performance** | **234 (4 RCT)** | **Some serious** | **Serious** | **Not serious** | **Some serious** | **None** | **0.63 (-0.09 to 1.35)** | **⨁◯◯◯ Very low** |
| **Agility Performance** | **302 (4RCT)** | **Some serious** | **Some serious** | **Not serious** | **Some serious** | **None** | **-0.24 (-0.82 to 0.33)** | **⨁◯◯◯ Very low** |
| **Off-ball sprint speed** | **138 (3 RCT)** | **Some serious** | **Not serious** | **Not serious** | **Some serious** | **None** | **-0.55 (-1.11 to 0.00)** | **⨁⨁◯◯ Low** |
| **Output Power** | **624 (2 RCT)** | **Some serious** | **Not serious** | **Not serious** | **Some serious** | **None** | **0.50 (-0.22 to 1.23)** | **⨁⨁◯◯ Low** |
| **Anaerobic Power** | **368 (3 RCT)** | **Some serious** | **Serious** | **Not serious** | **Some serious** | **None** | **-0.04 (-0.80 to 0.71)** | **⨁◯◯◯ Very low** |
| **Fatigue-Perception Performance** | **226 (4 RCT)** | **Some serious** | **Not serious** | **Not serious** | **Some serious** | **None** | **0.12 (-0.32 to 0.56)** | **⨁⨁◯◯ Low** |
| **Physiological and Biochemical markers** | **432 (2 RCT)** | **Some serious** | **Some serious** | **Not serious** | **Some serious** | **None** | **0.53 (-0.01 to 1.07)** | **⨁◯◯◯ Very low** |
| ***Certainty of evidence according to Grading of Recommendation, Assessment, Development, and Evaluations (GRADE):**  **Hight: We are very confidence in the estimated effect**  **Moderate: Our confidence in the estimated effect is moderate**  **Low: We have limited confidence in the estimate effect**  **Very low: We have very limited confidence in the estimate of effect**  **No of participants: Total number of participants with pooled effects** | | | | | | | | |

**Table note:** Publication bias was not formally assessed due to the small number of included studies (<10 per outcome). Consequently, the certainty of evidence was not downgraded for this domain; however, the limited number of studies reduces the statistical power to detect potential publication bias and should be considered a limitation of the evidence.

# Supplementary Table 6. Leave-One-Out Sensitivity Analysis: Influence of Individual Studies on Pooled Effect Estimates

| **Study Omitted** | **Number of Remaining Studies** | **SMD(95% CI)** | **p-value** | **I² (%)** | **Direction Consistent** | **Significance Change** |
| --- | --- | --- | --- | --- | --- | --- |
| **Jump** |  |  |  |  |  |  |
| **None** | 4 | 0.63 (−0.09, 1.35) | 0.08 | 77.2 | N/A | N/A |
| **Stojanovic et al, 2019** | 3 | 0.81 (-0.24, 1.85) | 0.111 | 83.9 | No | No |
| **Aleksandra et al, 2022** | 3 | 0.68 (-0.12, 1.16) | 0.088 | 79.5 | No | No |
| **Quan et al, 2024** | 3 | 0.56 (-0.45, 1.58) | 0.23 | 83.4 | No | No |
| **Raúl Nieto et al, 2025** | 3 | 0.54 (0.11, 0.96) | 0.022 | 0 | No | No |
|  |  |  |  |  |  |  |
|  |  |  |  |  |  |  |
| **Agility** |  |  |  |  |  |  |
| **None** | 4 | −0.24 (−0.82, 0.33) | 0.376 | 54.9 | N/A | N/A |
| **Stojanovic et al, 2019** | 3 | -0.24 (-0.88, 0.39) | 0.428 | 56.8 | No | No |
| **Aleksandra et al, 2022** | 3 | -0.27 (-0.90, 0.36) | 0.376 | 56.8 | No | No |
| **Quan et al, 2024** | 3 | -0.29 (-1.39, 0.81) | 0.369 | 0 | No | No |
| **Raúl Nieto et al, 2025** | 3 | -0.20 (-0.83, 0.44) | 0.518 | 56.3 | No | No |
|  |  |  |  |  |  |  |
|  |  |  |  |  |  |  |
| **sprint** |  |  |  |  |  |  |
| **None** | 3 | −0.55 (−1.11, 0.00) | 0.051 | 0 | N/A | N/A |
| **Stojanovic et al, 2019** | 2 | -0.59(-1.51, 0.32) | 0.129 | 0 | No | No |
| **Aleksandra et al, 2022** | 2 | -0.54 (-1.42, 0.35) | 0.148 | 0 | No | No |
| **Raúl Nieto et al, 2025** | 2 | -0.53 (-1.22, 0.17) | 0.108 | 0 | No | No |
|  |  |  |  |  |  |  |
|  |  |  |  |  |  |  |
| **Anaerobic Power** |  |  |  |  |  |  |
| **None** | 3 | −0.04 (−0.80, 0.71) | 0.896 | 86.6 | N/A | N/A |
| **Reza Mahdav et al, 2015** | 2 | 0.22 (-1.07, 1.50) | 0.631 | 43.2 | **Yes** | No |
| **Stojanovic et al, 2019** | 2 | 0.02 (-0.98, 1.02) | 0.969 | 89.1 | **Yes** | No |
| **L. Quan et al, 2024** | 2 | -0.30 (-1.36, 0.76) | 0.515 | 90.8 | No | No |
|  |  |  |  |  |  |  |
|  |  |  |  |  |  |  |
| **Fatigue-Perception Performance** |  |  |  |  |  |  |
| **None** | 4 | 0.12 (−0.32, 0.56) | 0.55 | 10 | N/A | N/A |
| **Reza Mahdav et al, 2015** | 3 | 0.18(-0.17, 0.53) | 0.272 | 0 | No | No |
| **Stojanovic et al, 2019** | 3 | -0.01(-0.49, 0.46) | 0.944 | 0 | **Yes** | No |
| **Aleksandra et al, 2022** | 3 | 0.06(-0.44, 0.56) | 0.79 | 12.4 | No | No |
| **L. Quan et al, 2024** | 3 | 0.28(-0.71, 1.27) | 0.436 | 51 | No | No |

# Supplementary Figure 7. Forest plots for each outcome

## Supplementary Figure 7.1. Shooting Accuracy


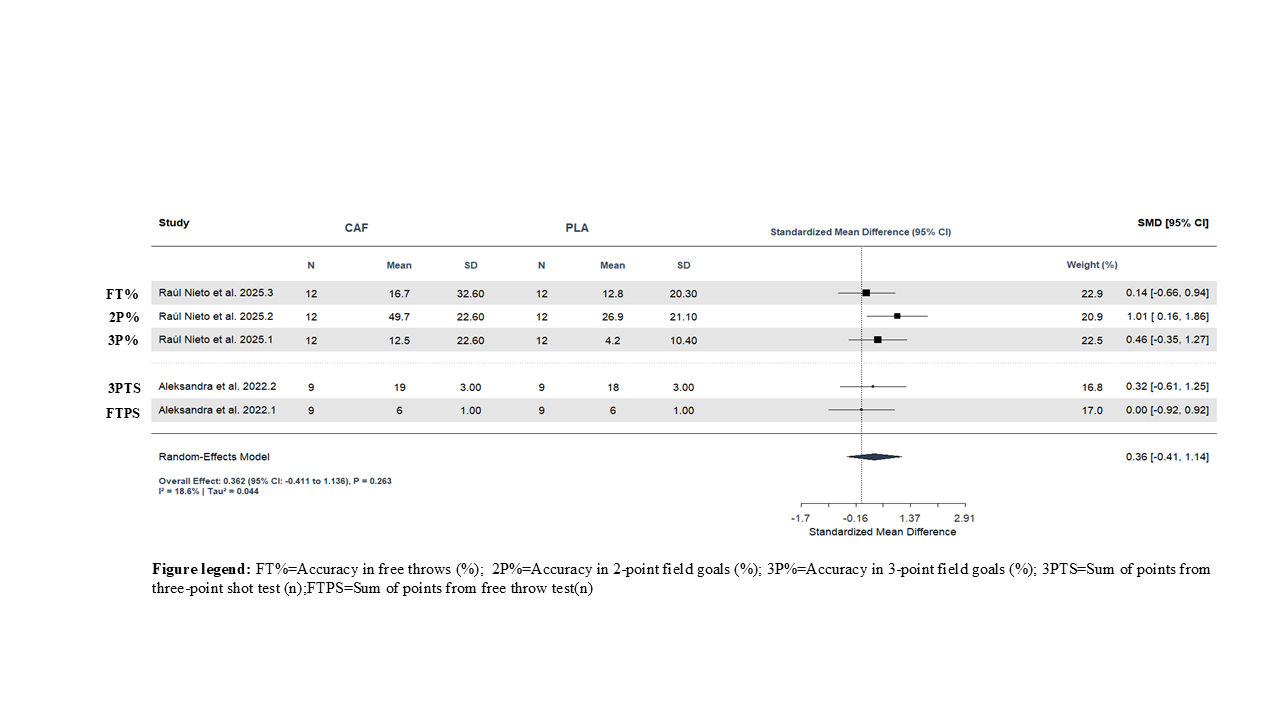


**Figure legend:** FT% = Accuracy in free throws (%)；2P% = Accuracy in 2-point field goals (%); 3P% = Accuracy in 3-point field goals (%); 3PTS = Sum of points from

three-point shot test (n); FTPS= Sum of points from free throw test (n).

##
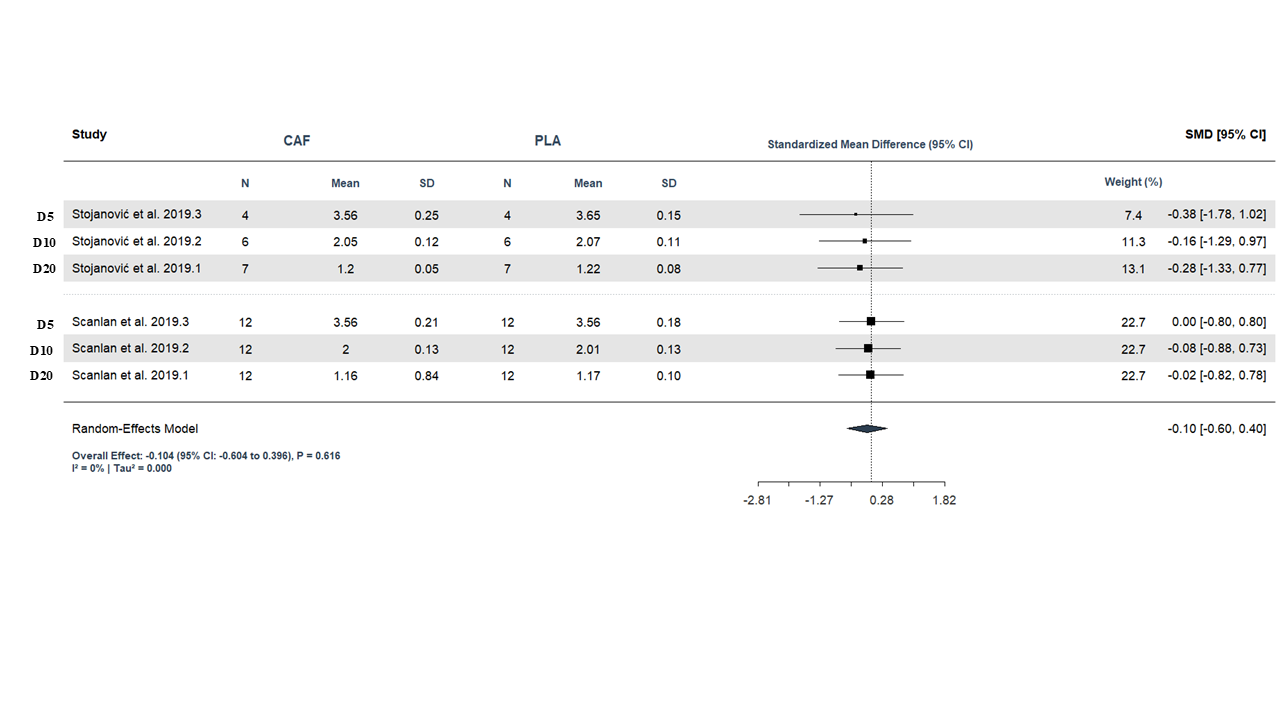
Supplementary Figure 7.2. Dribbling Speed

**Figure legend:** D5=5-m dribbling sprint (s)；D10=10-m dribbling sprint (s)；D20=20-m dribbling sprint (s).

## Supplementary Figure 7.3. Jump Performance


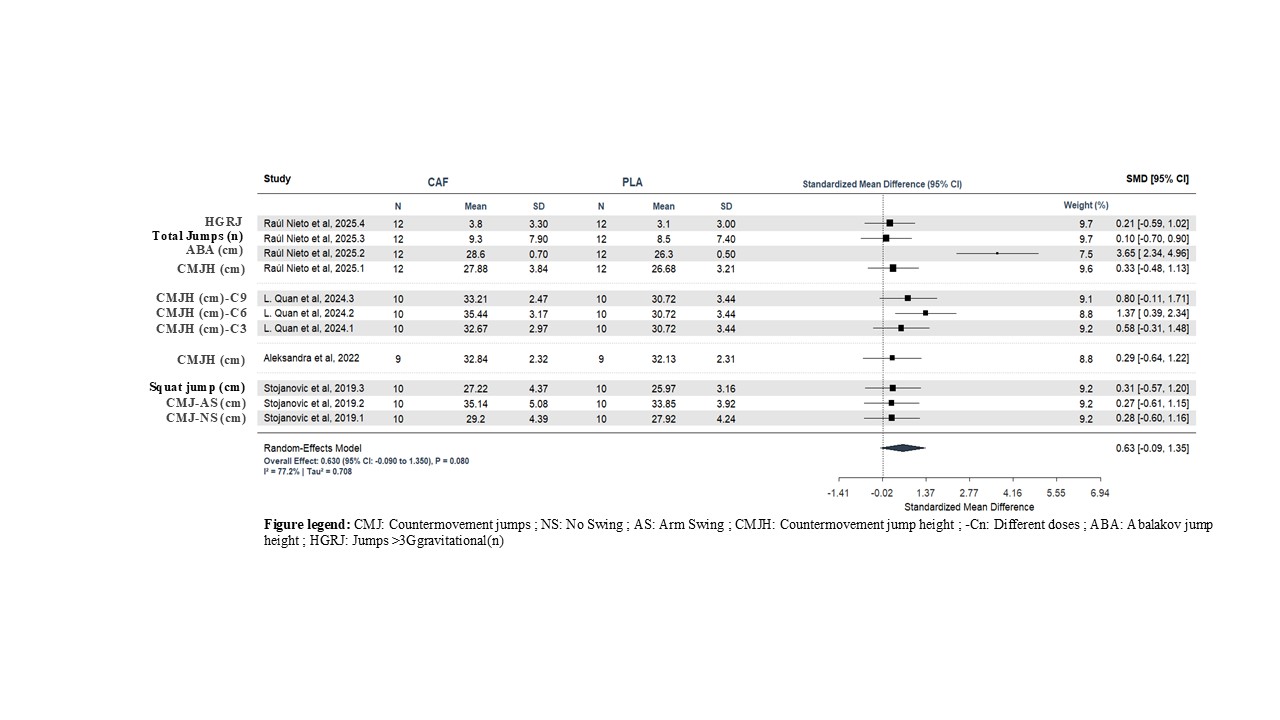


**Figure legend:** CMJ= Countermovement jumps; NS= No Swing; AS= Arm Swing; CMJH= Countermovement jump height; -Cn= Different doses; ABA=

Abalakov jump height; HGRJ= Jumps >3Ggravitational(n).

##
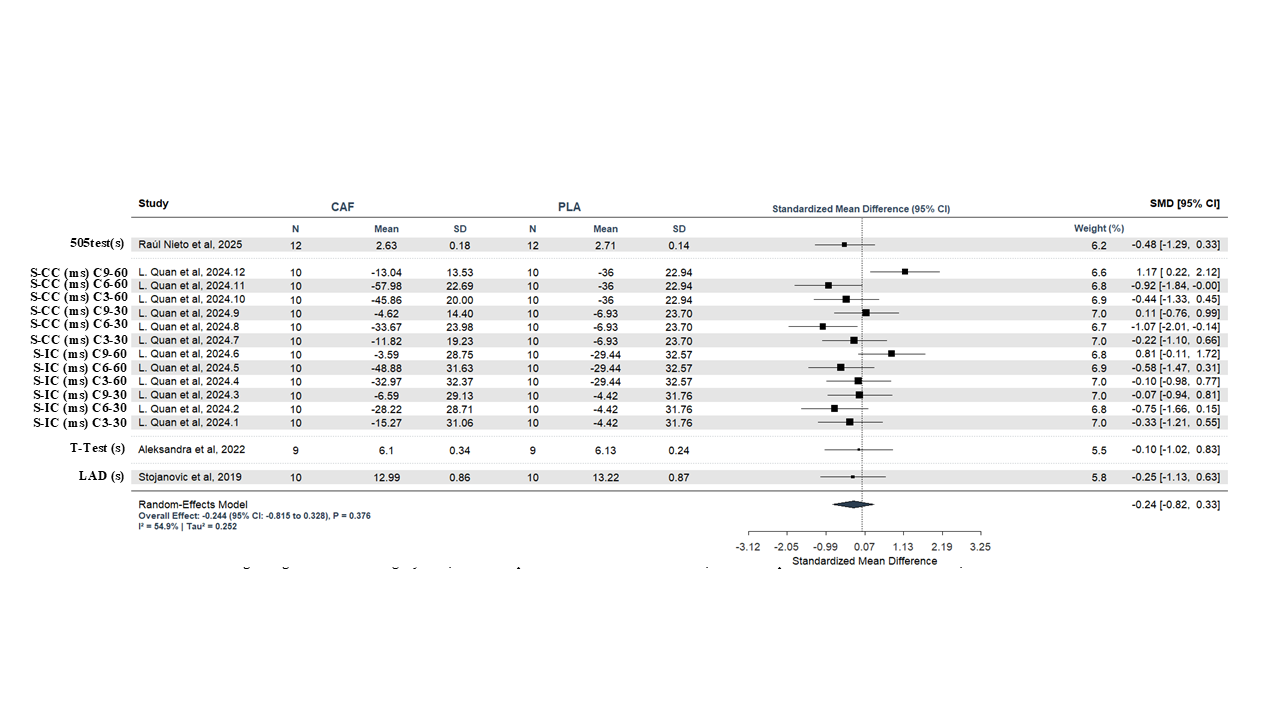
Supplementary Figure 7.4. Agility Performance

**Figure legend:** LAD=Lane Agility Drill; S-IC=Stroop test under inconsistent conditions; S-CC=Stroop test under consistent conditions; Cn-n=Dose and Administ-

ration time.

##
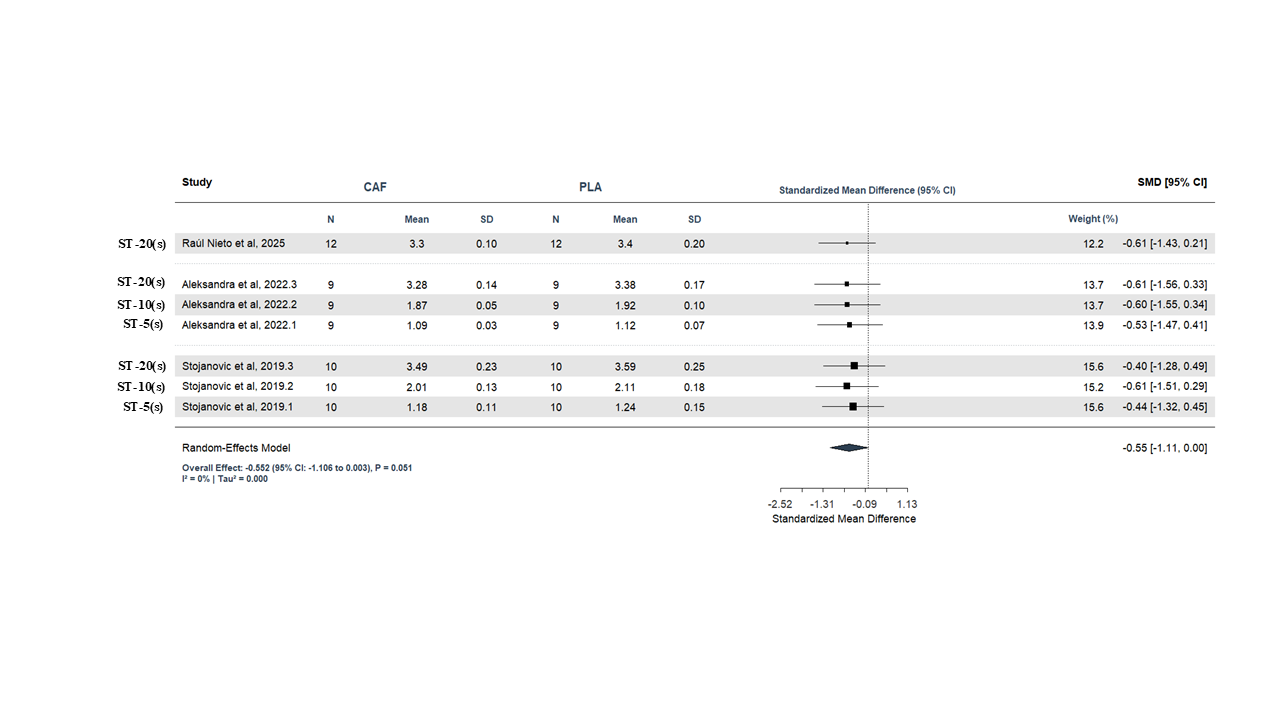
Supplementary Figure 7.5. Off-ball sprint speed

**Figure legend:** ST=Sprint Test; -n=meter; s=second

##
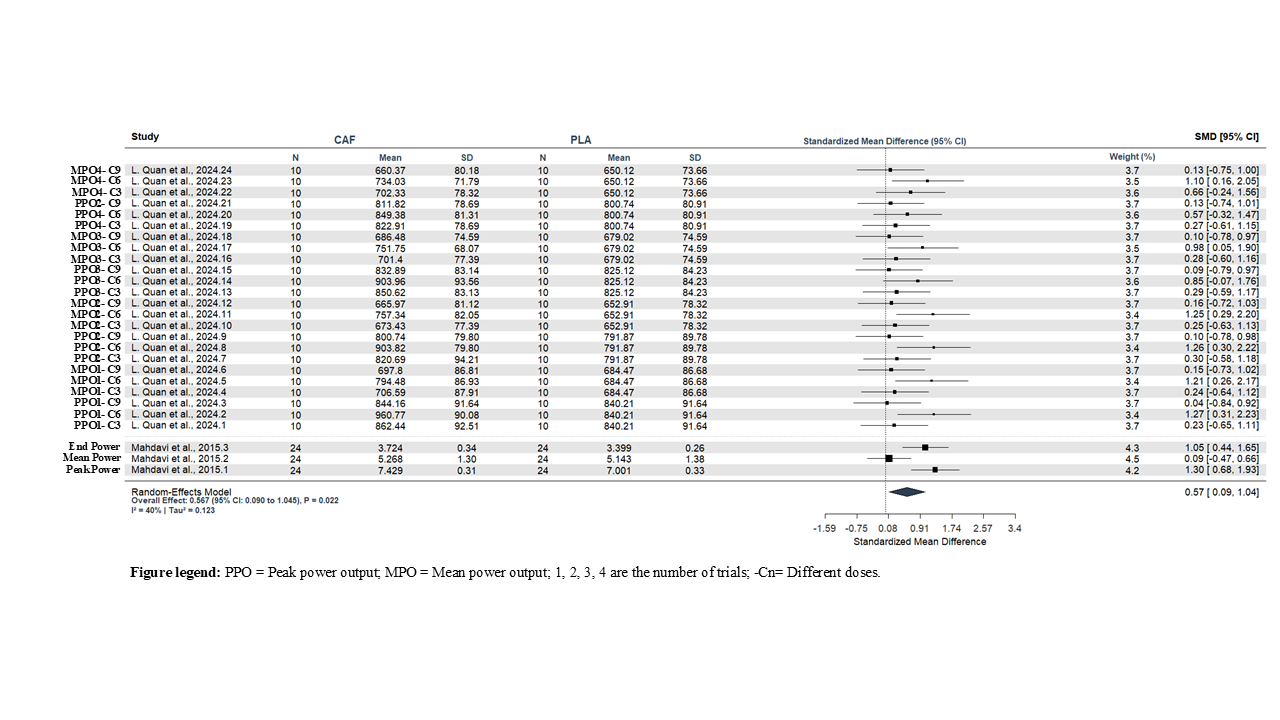
Supplementary Figure 7.6. Power Output

**Figure legend：**PPO = Peak power output; MPO = Mean power output; 1, 2, 3, 4 are the number of trials; -Cn= Different doses.

##
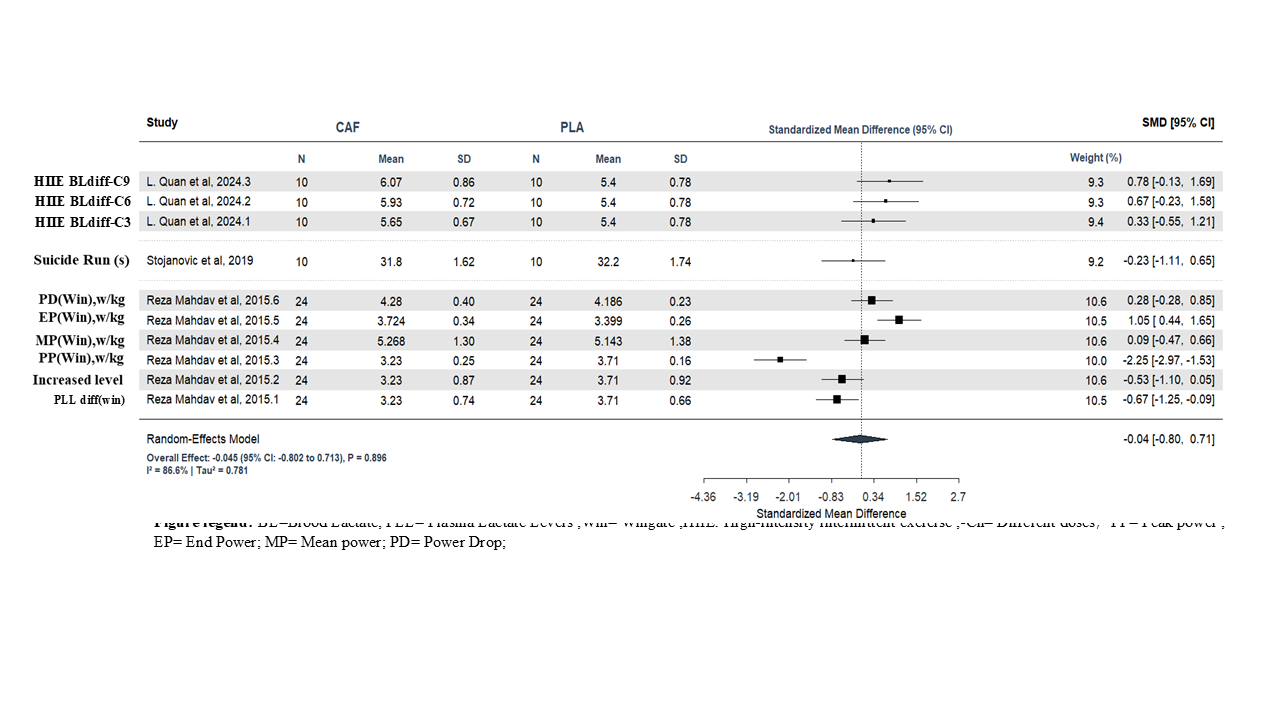
Supplementary Figure 7.7. Anaerobic Performance

**Figure legend:** BL=Blood Lactate; PLL= Plasma Lactate Levels ;Win= Wingate ;HIIE: High-intensity intermittent exercise ;-Cn= Different doses；PP= Peak power；

EP= End Power; MP= Mean power; PD= Power Drop.

##
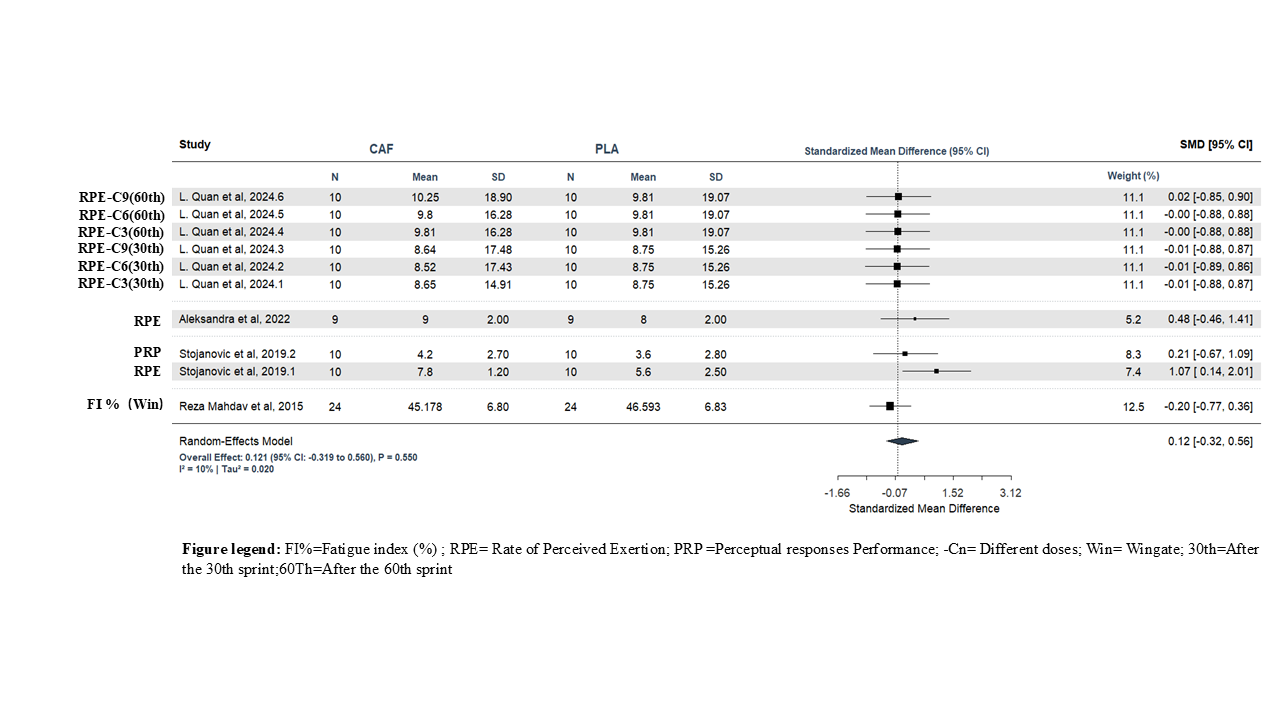
Supplementary Figure 7.8. Fatigue-Perception Performance

**Figure legend:** FI%=Fatigue index (%); RPE= Rate of Perceived Exertion; PRP =Perceptual responses Performance; -Cn= Different doses; Win= Wingate;

30th=After the 30th sprint;60Th=After the 60th sprint.

##
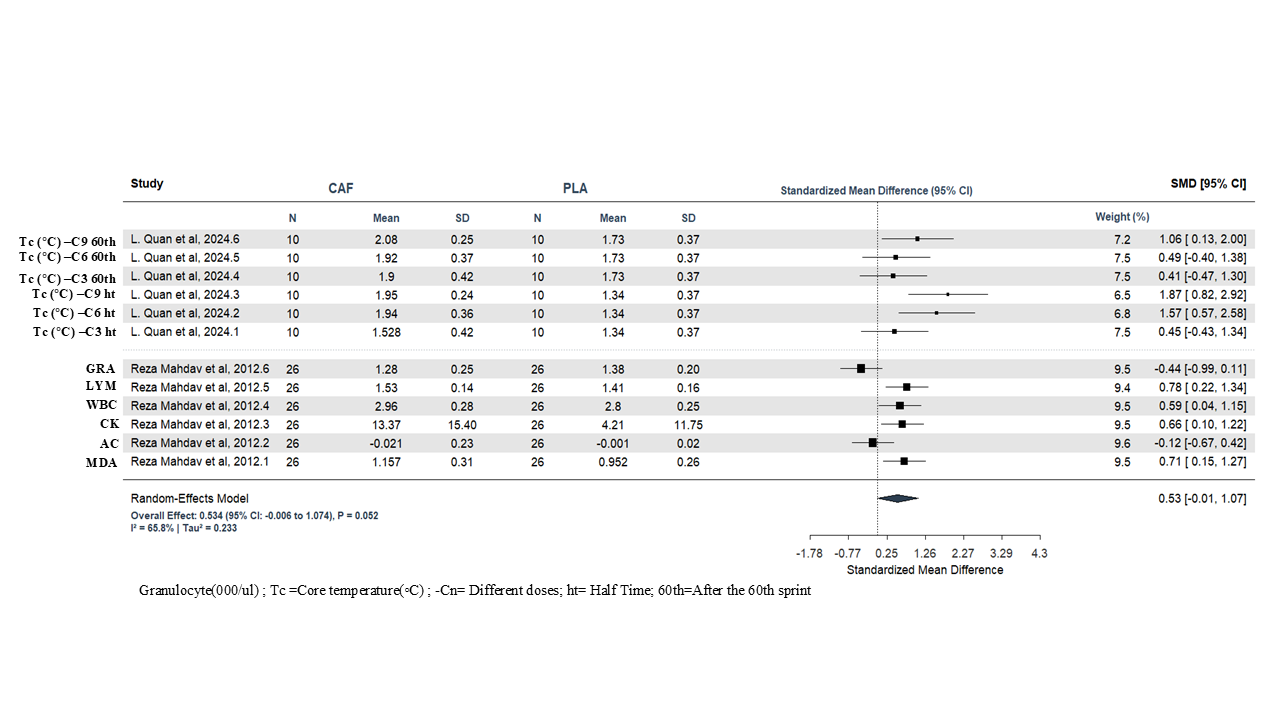
Supplementary Figure 7.9. Physiological and Biochemical markers

**Figure legend:** MDA=Malondialdehyde(nm/ml); AC=Antioxidant capacity(mmol/l); CK=Creatine kinase (IU/L); WBC=White blood cells(000/uL); LYM= Lymphocyte(000/ul);

GRA=Granulocyte(000/ul); Tc =Core temperature(◦C); -Cn= Different doses; ht= Half Time; 60th=After the 60th sprint.

# Supplementary Table 8. Subgroup Analyses

## Supplementary Table 8.1 Subgroup Analysis of Meta-Analysis Results for Jump Performance

| **Subgroup** | **K(N)** | **Hedges' *g*** | **95% CI** | ***p*_d_** | ***Q*** | ***I*^2^** | ***power*** | ***p*_m_** |
| --- | --- | --- | --- | --- | --- | --- | --- | --- |
| **Caffeine Dosage** |  |  |  |  |  |  |  | 0.38 |
| Low（≤3mg/kg） | 9 (194) | 0.56 | [0.01, 1.11] | 0.03 | 0.70 | 64% | 23% |  |
| Moderate(＞3-6mg/kg) | 1 (20) | 1.37 | [0.37, 2.36] | **＜0.01** | 0.00 | n/a | 82% |  |
| High（>6–≥9 mg/kg ） | 1 (20) | 0.80 | [-0.12, 1.71] | 0.09 | 0.00 | n/a | 39% |  |
| **Jump Classification** |  |  |  |  |  |  |  | **＜0.01** |
| Countermovement Jump | 7 (142) | 0.53 | [0.19, 0.87] | **＜0.01** | 4.17 | 0% | 20% |  |
| Abalakov Jump | 1 (24) | 3.65 | [2.27, 5.04] | 0.00 | 0.00 | n/a | 100% |  |
| Jump Capacity | 2 (48) | 0.16 | [-0.41, 0.72] | 0.59 | 0.04 | 0% | 7% |  |
| Squat Jump | 1 (20) | 0.31 | [-0.57, 1.20] | 0.49 | 0.00 | n/a | 10% |  |
| **Jump Functional Characteristics** |  |  |  |  |  |  |  |  |
| Maximal-Effort Jumping | 9 (182) | 0.77 | [0.24, 1.30] | **＜0.01** | 23.26 | 66% | 38% | 0.12 |
| Repeated-Effort Capacity | 3 (156) | 0.16 | [-0.41, 0.72] | 0. | 0.04 | 0% | 7% |  |
| **Jump Technical Complexity** |  |  |  |  |  |  |  | **＜0.01** |
| Lower-Limb Dominant Jump | 2 (40) | 0.30 | [-0.33, 0.92] | 0.35 | 0.00 | 0 | 10% |  |
| Basic Coordinative Jumps | 6 (122) | 0.57 | [0.20, 0.94] | **＜0.01** | 21.33 | 0% | 23% |  |
| Sport-Specific Skill Jumps | 1 (24) | 3.65 | [2.27, 5.04] | 0.00 | 21.33 | n/a | **100%** |  |
| **Jump Mechanical Objective** |  |  |  |  |  |  |  | 0.3 |
| Maximal Power Output | 9 (186) | 0.77 | [0.24, 1.30] | **＜0.01** | 23.26 | 66% | 38% |  |
| Mechanical Impulse | 1 (24) | 0.21 | [-0.59, 1.02] | 0.8 | 0.00 | n/a | 6% |  |
| Metabolic Power | 1(24) | 0.10 | [-0.70, 0.90] | 0.6 | 0.00 | n/a | 8% |  |
| **Risk of bias–based** |  |  |  |  |  |  |  | 0.49 |
| Low risk | 8 (174) | 0.58 | [-0.08, 1.25] | 0.05 | 22.30 | 69 | 23% |  |
| Some/High risk | 3 (60) | 0.89 | [ 0.35, 1.43] | **＜0.01** | 1.38 | 0 | 47% |  |
| **Training level** |  |  |  |  |  |  |  | 052 |
| Experience /Professional/Elite | 7 (138) | 0.53 | [ 0.19, 0.88] | **＜0.01** | 4.15 | 0 | 20 |  |
| semi-professional | 4 (96) | 0.95 | [-0.26, 2.16] | 0.12 | 21.44 | 86 | 60 |  |

**Table note:** K(N) = number of included effect sizes (total number of combined subjects); A positive value of the combined effect size (Hedges' g) indicates that the caffeine group improved jump performance compared to the placebo group; pd = p-value of the combined effect size; power = statistical power of the subgroup combined result; pm = p-value for the difference be-tween subgroups.

| **Subgroup** | **K(N)** | **Hedges' *g*** | **95% CI** | ***p*_d_** | ***Q*** | ***I*^2^** | ***power*** | ***p*_m_** |
| --- | --- | --- | --- | --- | --- | --- | --- | --- |
| **Caffeine Dosage** |  |  |  |  |  |  |  | **＜0.01** |
| Low（≤3mg/kg） | 7 (142) | -0.28 | [-0.61, 0.05] | 0.1 | 0.69 | 0% | 9% |  |
| Moderate (>3–6 mg/kg) | 4 (80) | -0.82 | [-1.29, -0.36] | ＜0.01 | 0.61 | 0% | 42% |  |
| High（>6–≥9 mg/kg） | 4 (80) | 0.48 | [-0.08, 1.04] | 0.1 | 4.60 | 35% | 17% |  |
| **Unit of Measurement** |  |  |  |  |  |  |  | 0.77 |
| Second | 3 (62) | -0.29 | [-0.80, 0.21] | 0.25 | 0.38 | 0% | 10% |  |
| Millisecond | 12 (240) | -0.20 | [-0.56, 0.16] | 0.27 | 20.96 | 48% | 7% |  |
| **Mental Workload Intensity** |  |  |  |  |  |  |  | 0.8 |
| Low Cognitive Demand Tasks | 9 (182) | -0.25 | [-0.65, 0.15] | 0.22 | 14.41 | 44% | 8% |  |
| High Cognitive Demand Tasks | 3 (156) | -0.17 | [-0.60, 0.25] | 0.43 | 6.88 | 27% | 7% |  |
| **Form of Caffeine Administration** |  |  |  |  |  |  |  | 0.79 |
| Gum | 1 (18) | 0.80 | [0.44, 1.41] | 0.84 | 0.00 | n/a | 5% |  |
| Capsules | 1 (284) | -0.23 | [-0.54, 0.08] | 0.14 | 21.33 | 39% | 8% |  |
| **Task Openness** |  |  |  |  |  |  |  | 0.82 |
| Closed Skills | 12 (240) | -0.20 | [-0.56, 0.16] | 0.27 | 20.993 | 47% | 7% |  |
| Intermediate Skills | 1 (24) | -0.48 | [-1.29, 0.33] | 0.25 | 0.00 | n/a | 20% |  |
| Open Skills | 2 (38) | -0.18 | [-0.82, 0.46] | 0.58 | 0.06 | 0% | 7% |  |
| **Risk of bias–based** |  |  |  |  |  |  |  | 0.77 |
| Low risk | 3 (62) | -0.29 | [-0.80, 0.21] | 0.10 | 0.38 | 0 | 10 |  |
| Some/High risk | 12 (240) | -0.20 | [-0.56, 0.16] | 0.27 | 20.93 | 47% | 7 |  |
| **Training level** |  |  |  |  |  |  |  | 0.53 |
| Experience /Professional/Elite | 14 (278) | -0.20 | [-0.51, 0.11] | 0.20 | 20.99 | 38% | 7 |  |
| semi-professional | 1 (24) | -0.48 | [-1.29, 0.33] | 0.24 | 0.00 | n/a | 20 |  |

## Supplementary Table 8.2 Subgroup Analysis of Meta-Analysis Results for Agility

**Table note:** K(N) = number of included effect sizes (total number of combined subjects); A negative value for the combined effect size (Hedges' g) indicates that caffeine improves reactive agility performance compared to the placebo; pd = p-value of the combined effect size; power = statistical power of the subgroup combined result; pm = p-value for the difference between subgroups.

## Supplementary Table 8.3 Subgroup Analysis of Meta-Analysis Results for Power Output

| **Subgroup** | **K(N)** | **Hedges' *g*** | **95% CI** | ***p*_d_** | ***Q*** | ***I*^2^** | ***power*** | ***p*_m_** |
| --- | --- | --- | --- | --- | --- | --- | --- | --- |
| **Caffeine Dosage** |  |  |  |  |  |  |  | **＜0.01** |
| Low（≤3mg/kg） | 8 (160) | 0.31 | [0.001, 0.63] | 0.05 | 0.65 | 0% | 10% |  |
| Moderate (>3–6 mg/kg) | 11 (304) | 0.93 | [ 0.66, 1.21 ] | **＜0.01** | 12.32 | 19% | 66% |  |
| High（>6–≥9 mg/kg ） | 8 (160) | 0.11 | [-0.20, 0.42] | 0.48 | 0.05 | 0% | 6% |  |
| **Outcome metric type** |  |  |  |  |  |  |  | 0.18 |
| Peak power | 13 (288) | 0.53 | [ 0.25, 0.82 ] | **＜0.01** | 15.85 | 24% | 22% |  |
| Mean power | 13 (288) | 0.44 | [ 0.20, 0.68 ] | **＜0.01** | 12.25 | 2% | 16% |  |
| End power | 1 (48) | 1.05 | [ 0.44, 1.65] | **＜0.01** | 0.00 | n/a | 94% |  |
| **Test modality** |  |  |  |  |  |  |  | 0.38 |
| Wingate | 3 (144) | 0.80 | [ 0.07, 1.54 ] | 0.03 | 9.10 | 78% | 78% |  |
| Intermittent | 24 (480) | 0.46 | [ 0.28, 0.65 ] | **＜0.01** | 20.06 | 0% | 17% |  |
| **Training level** |  |  |  |  |  |  |  | 0.38 |
| Experience | 24 (480) | 0.46 | [ 0.28, 0.65 ] | **＜0.01** | 20.06 | 0% | 17% |  |
| Not | 3 (144) | 0.80 | [ 0.07, 1.54] | 0.03 | 9.10 | 78% | 78 |  |

**Table note:** K(N) = number of included effect sizes (total number of combined subjects); A positive pooled Hedges' g value indicates that caffeine has an improving effect on the physiological and biochemical indicators compared to placebo；pd = p-value of the combined effect size; power = statistical power of the subgroup combined result; pm = p-value for the difference between subgroups.

| **Subgroup** | **K(N)** | **Hedges' *g*** | **95% CI** | ***p*_d_** | ***Q*** | ***I*^2^** | ***power*** | ***p*_m_** |
| --- | --- | --- | --- | --- | --- | --- | --- | --- |
| **Caffeine Dosage** |  |  |  |  |  |  |  | 0.25 |
| Low（≤3mg/kg） | 2 (40) | 0.05 | [-0.93,0.54] | 0.88 | 0.76 | 0% | 5% |  |
| Moderate (>3–6 mg/kg) | 7(308) | -0.19 | [ -0.93, 0.54] | 0.61 | 57.11 | 89% | 10% |  |
| High（>6–≥9 mg/kg ） | 1(20) | 0.78 | [-0.14, 1.70] | 0.10 | 0.00 | n/a | 38% |  |
| **Direction of Beneficial Effect** |  |  |  |  |  |  |  | 0.13 |
| Negative Direction Indicator | 3 (116) | -0.53 | [-0.90, -0.16] | **＜0.01** | 0.68 | 0% | 37% |  |
| Positive direction indicators | 7 (252) | 0.13 | [-0.67, 0.93] | 0.75 | 52.81 | 89% | 7% |  |
| **Measurement Method** |  |  |  |  |  |  |  | 0.41 |
| Direct Measurement Method | 6 (260) | -0.25 | [-1.08, 0.58] | 0.56 | 50.88 | 90% | 13% |  |
| Blood Lactate Measurement | 4 (108) | 0.22 | [-0.54, 0.98] | 0.57 | 10.53 | 72% | 9% |  |
| **Physiological Function** |  |  |  |  |  |  |  | 0.3 |
| Anaerobic Power Output | 2 (96) | -1.07 | [-3.36, 1.23] | 0.36 | 24.45 | 96% | 95% |  |
| Metabolic Reaction | 5 (156) | 0.04 | [-0.57, 0.64] | 0.9 | 12.99 | 69% | 5% |  |
| Comprehensive Athletic Performance | 1 (20) | -0.23 | [-1.11, 0.65] | 0.61 | 0.00 | n/a | 8% |  |
| Fatigue and Recovery | 2 (96) | 0.66 | [-0.09, 1.40] | 0.08 | 3.23 | 69% | 60% |  |
| **Dominant Metabolic Energy System** |  |  |  |  |  |  |  | **＜0.01** |
| Phosphagen System | 1 (48) | -2.25 | [-2.98, -1.51] | 0.00 | n/a | n/a | 100% |  |
| Glycolytic System | 8 (300) | 0.21 | [-0.24, 0.66] | 0.35 | 24.86 | 72% | 10% |  |
| Mixed & Comprehensive | 1 (20) | -0.23 | [-1.11, 0.65] | 0.61 | n/a | n/a | 8% |  |
| **Exercise Testing Mode** |  |  |  |  |  |  |  | 0.87 |
| Wingate Anaerobic Test | 4 (192) | -0.19 | [-1.42, 1.04] | 0.76 | 48.45 | 94% | 10% |  |
| Blood Lactate Response | 5 (156) | 0.04 | [-0.57, 0.64] | 0.61 | 12.99 | 69% | 5% |  |
| Comprehensive Field Test | 1 (20) | -0.23 | [-1.11, 0.65] | 0.9 | 0.00 | n/a | 8% |  |
| **Statistical Properties of the Outcomes** |  |  |  |  |  |  |  |  |
| Absolute Measurements | 2 (96) | -1.07 | [-3.36, 1.23] | 0.36 | 24.45 | 96% | 95% | 0.3 |
| Calculated Ratios/Percentages | 2 (96) | 0.66 | [-0.09, 1.40] | 0.08 | 3.23 | 69% | 61% |  |
| Change-from-Baseline Scores | 5 (156) | 0.04 | [-0.57, 0.64] | 0.9 | 12.99 | 69% | 5% |  |
| Composite Task Scores | 1 (20) | -0.23 | [-1.11, 0.65] | 0.61 | 0.00 | n/a | 8% |  |
| **Risk of bias–based** |  |  |  |  |  |  |  | 0.73 |
| Low risk | 1 (20) | -0.23 | [-1.11, 0.65] | 0.90 | 0.00 | n/a | 8% |  |
| Some/High risk | 9 (348) | -0.04 | [-0.67, 0.58] | 0.61 | 61.77 | 87% | 5% |  |
| **Training level** |  |  |  |  |  |  |  | 0.14 |
| Experience /Professional | 4 (80) | 0.38 | [-0.07, 0.83] | 0.10 | 53.61 | 0 | 13 |  |
| Not | 6(288) | -0.32 | [-1.13, 0.48] | 0.43 | 2.98 | 91% | 20 |  |

## Supplementary Table 8.4 Subgroup Analysis of Meta-Analysis Results for Anaerobic Performance

**Table note:** K(N) = number of included effect sizes (total number of combined subjects); The interpretation of Hedges' g depends on the metric: positive values indicate caffeine benefits when higher scores reflect improvement (e.g., power); negative values indicate benefits when higher scores reflect impairment (e.g., completion time) ；pd = p-value of the combined effect size; power = statistical power of the subgroup combined result; pm = p-value for the difference between subgroups.

## Supplementary Table 8.5 Subgroup Analysis of Meta-Analysis Results for Physiological and Biochemical markers

| **Subgroup** | **K(N)** | **Hedges' *g*** | **95% CI** | ***p*_d_** | ***Q*** | ***I*^2^** | ***power*** | ***p*_m_** |
| --- | --- | --- | --- | --- | --- | --- | --- | --- |
| **Caffeine Dosage** |  |  |  |  |  |  |  | 0.09 |
| Low（≤3mg/kg） | 2 (56) | 0.44 | [-0.19, 1.06] | 0.17 | 0 | 0% | 15% |  |
| Moderate (>3–6 mg/kg) | 8 (176) | 0.47 | [ 0.08, 0.86] | 0.02 | 21.55 | 68% | 33% |  |
| High（>6–≥9 mg/kg ） | 2 (20) | 1.43 | [0.63, 2.23] | ＜0.01 | 1.23 | 19% | 86% |  |
| **Direction of Beneficial Effect** |  |  |  |  |  |  |  | **0.01** |
| Negative Direction Indicator | 8 (224) | 0.81 | [0.5, 1.12] | n/a | 8.37 | 16% | 54% |  |
| Positive direction indicators | 1 (52) | -0.12 | [-0.67,0.42] | 0.66 | n/a | n/a | 7% |  |
| Neutral Indicators | 3 (156) | 0.31 | [-0.43, 1.05] | 0.41 | 10.68 | 81% | 20% |  |
| **Physiological type** |  |  |  |  |  |  |  | 0.25 |
| Oxidative Stress and Damage Markers | 3 (156) | 0.41 | [-0.12, 0.94] | **0.13** | 5.51 | 64% | 31% |  |
| Immune and Inflammatory Markers | 3 (156) | 0.31 | [-0.43, 1.05] | 0.41 | 10.68 | 81% | 20% |  |
| Thermoregulation | 6 (120) | 0.92 | [0.44, 1.41] | ＜0.01 | 7.81 | 36% | 50% |  |
| **Sampling Method** |  |  |  |  |  |  |  | 0.08 |
| Tympanic Thermometer | 6 (120) | 0.80 | [0.44, 1.41] | **＜0.01** | 7.81 | 36% | 50% |  |
| Blood Sampling | 6 (312) | 0.11 | [-0.05, 0.77] | 0.09 | 16.39 | 39% | 25% |  |
| **Risk of bias–based** |  |  |  |  |  |  |  |  |
| Some risk | 6 (312) | 0.36 | [-0.05, 0.77] | 0.09 | 16.39 | 69% | 25% | 0.83 |
| High risk | 6 (120) | 0.92 | [ 0.44, 1.41] | **＜0.01** | 7.81 | 36% | 50% |  |

**Table note:** K(N) = number of included effect sizes (total number of combined subjects); A positive pooled Hedges' g value indicates that caffeine has an improving effect on the physiological and biochemical indicators compared to placebo；pd = p-value of the combined effect size; power = statistical power of the subgroup combined result; pm = p-value for the difference between subgroups.

# Supplementary Table 9. Sensitivity analyses based on prespecified criteria

| Outcome domain | Analysis | Effect sizes included (N) | SMD (95% CI) | I² (%) | Interpretation |
| --- | --- | --- | --- | --- | --- |
| Jump performance | Main analysis | 11 | 0.63 (-0.09, 1.35） | 77.2 |  |
|  | Excluding Domain 5 studies | 8 | 0.56 (-0.45, 1.58) | 83.4 | Direction unchanged |
| Agility | Main analysis | 15 | −0.24 (−0.82, 0.33) | 54.9 |  |
|  | Excluding Domain 5 studies | 3 | -0.38 (-1.50, 0.74) | 0 | Direction unchanged |
| Power Output | Main analysis | 27 | 0.57 ( 0.09, 1.04 ) | 40 |  |
|  | Excluding Domain 5 studies | 3 | 0.80 (-1.04, 2.65) | 79.7 | Direction unchanged |
| Anaerobic Power | Main analysis | 10 | −0.04 (−0.80, 0.71) | 86.6 |  |
|  | Excluding Domain 5 studies | 1 | -0.23 (-1.11, 0.65) | N/A | Direction unchanged |
| Physiological and Biochemical markers | Main analysis | 12 | 0.53 (−0.01, 1.07) | 65.8 |  |
|  | Excluding Domain 5 studies | 0 | Not estimable | N/A | Not estimable |

**Table note:** SMD= standardized mean difference; CI= confidence interval; I²= between-study heterogeneity

N= the number of effect sizes included in each analysis.
